# Synergistic photothermal-photodynamic-chemotherapy toward breast cancer based on a liposome-coated core–shell AuNS@NMOFs nanocomposite encapsulated with gambogic acid

**DOI:** 10.1186/s12951-022-01427-4

**Published:** 2022-05-06

**Authors:** Rong-Tian Li, Yi-Dan Zhu, Wen-Ya Li, Ying-Ke Hou, Yi-Ming Zou, Ying-Hua Zhao, Quan Zou, Wen-Hua Zhang, Jin-Xiang Chen

**Affiliations:** 1 NMPA Key Laboratory for Research and Evaluation of Drug Metabolism, Guangdong Provincial Key Laboratory of New Drug Screening, School of Pharmaceutical Sciences, Southern Medical University, Guangzhou, 510515 People’s Republic of China; 2 College of Chemistry, Chemical Engineering and Materials Science, Soochow University, Suzhou, 215123 People’s Republic of China; 3 Department of Medical Imaging, Third Affiliated Hospital of Southern Medical University (Academy of Orthopedics Guangdong Province), Southern Medical University, Guangzhou, 510630 People’s Republic of China

**Keywords:** Gold nanostar, Nanoscale metal−organic framework, Gambogic acid, Photothermal therapy, Photodynamic therapy, Chemotherapy

## Abstract

A multifunctional nanoplatform with core–shell structure was constructed in one-pot for the synergistic photothermal, photodynamic, and chemotherapy against breast cancer. In the presence of gambogic acid (GA) as the heat-shock protein 90 (HSP90) inhibitor and the gold nanostars (AuNS) as the photothermal reagent, the assembly of Zr^4+^ with tetrakis (4-carboxyphenyl) porphyrin (TCPP) gave rise to the nanocomposite AuNS@ZrTCPP-GA (**AZG**), which in turn, further coated with PEGylated liposome (LP) to enhance the stability and biocompatibility, and consequently the antitumor effect of the particle. Upon cellular uptake, the nanoscale metal − organic framework (NMOF) of ZrTCPP in the resulted AuNS@ZrTCPP-GA@LP (**AZGL**) could be slowly degraded in the weak acidic tumor microenvironment to release AuNS, Zr^4+^, TCPP, and GA to exert the synergistic treatment of tumors via the combination of AuNS-mediated mild photothermal therapy (PTT) and TCPP-mediated photodynamic therapy (PDT). The introduction of GA serves to reduce the thermal resistance of the cell to re-sensitize PTT and the constructed nanoplatform demonstrated remarkable anti-tumor activity in vitro and in vivo. Our work highlights a facile strategy to prepare a pH-dissociable nanoplatform for the effective synergistic treatment of breast cancer.

**Graphical Abstract:**

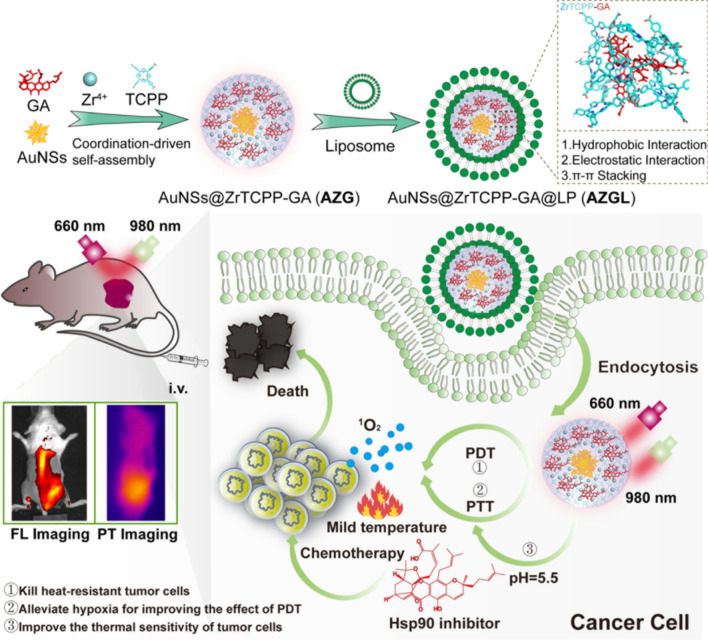

**Supplementary Information:**

The online version contains supplementary material available at 10.1186/s12951-022-01427-4.

## Introduction

As an effective and non-invasive cancer treatment, phototherapy has attracted increasing attention due to its advantages of nondrug resistance and low toxicity [1]. Phototherapy is mainly represented by photodynamic therapy (PDT) and photothermal therapy (PTT). PDT is based on photosensitive drugs to transfer the absorbed energy to the surrounding oxygen and generate cytotoxic reactive oxygen species (ROS) [2], whereas PTT relies on materials with light-to-heat conversion ability to kill cancer cells upon preferably near-infrared (NIR) light irradiation [3].

In the past decade, many photosensitizers, such as porphyrins, chlorins, and phthalocyanines have been raised an increased interest for their PDT effect in cancer therapy [4–6]. However, these organic species bearing extended π systems readily aggregate to give low water solubility and poor ROS generation efficacy. To overcome these drawbacks, researchers have used these organic photosensitizers to assemble with metal ions to construct nanoscale metal−organic frameworks (NMOFs) to enhance their stability and dispersity [7–10]. Among those organic photosensitizers, special attention was paid to the porphyrin derivative TCPP [TCPP = tetrakis (4-carboxyphenyl) porphyrin] due to its merits of fluorescence imaging and PDT effect [11, 12]. The appropriate proportion of metal ions and TCPP can result in the reasonable force between the metal ions and carboxylate groups to disrupt the π–π stacking of the free TCPP ligands and form NMOFs with good biological and light stability [13, 14].

On the other hand, among various photothermal nanomaterials, gold-based nanoparticles have attracted much recent attention on account of their controllable morphology and high photothermal conversion efficiency [15, 16], with gold nanostars (AuNS) featuring multiple sharp tips particularly attractive due to their strong NIR absorption accompanied by a tunable photothermal response to light and their simple preparation, low toxicity, and good biocompatibility [17]. It was reported that the combination of the photothermal treatment of AuNS and the tumor-targeting effect in tumor cell-derived stellate plasmonic exosomes (TDSP-Exos) give rise to excellent anti-tumor activity [18].

As a local therapeutic regimen, either PDT or PTT alone has shown satisfying therapeutic efficacy in vivo [19]. In recent years, it has been found that the synergistic operation of PDT and PTT can significantly enhance the anti-tumor response [20, 21]. For example, the nanocomposite constructed from Prussian blue and porphyrin-based NMOFs with both PDT and PTT characteristics showed an improved therapeutic outcome as compared to monotherapy [22]. The photothermal agents generate heat to promote blood circulation to enhance the oxygen supply to the tumor microenvironment to strengthen the effect of PDT. In a complementary manner, the ROS produced by PDT kills heat-resistant tumor cells to improve the effect of PTT [23]. Therefore, PDT and PTT can be operated synergistically to maximize the therapeutic outcome.

It is established that the temperature of the tumor area must be higher than 50 °C for effective tumor ablation during PTT [24]. Nevertheless, such a high temperature is expected to damage the surrounding normal tissues. Mild hyperthermia (below 45 °C) arising from PTT could effectively kill tumor cells for a longer time, but tumor cells will over-express heat shock proteins (HSPs) to protect themselves from heat damage and resist PTT after a certain time [25]. The latest research has demonstrated that gambogic acid (GA) has a highly effective inhibitory effect on the heat-shock protein 90 (HSP90) to effectively restore the sensitivity of cells to heat [26]. However, its high toxicity severely limited its clinical adoption.

**Scheme 1:**
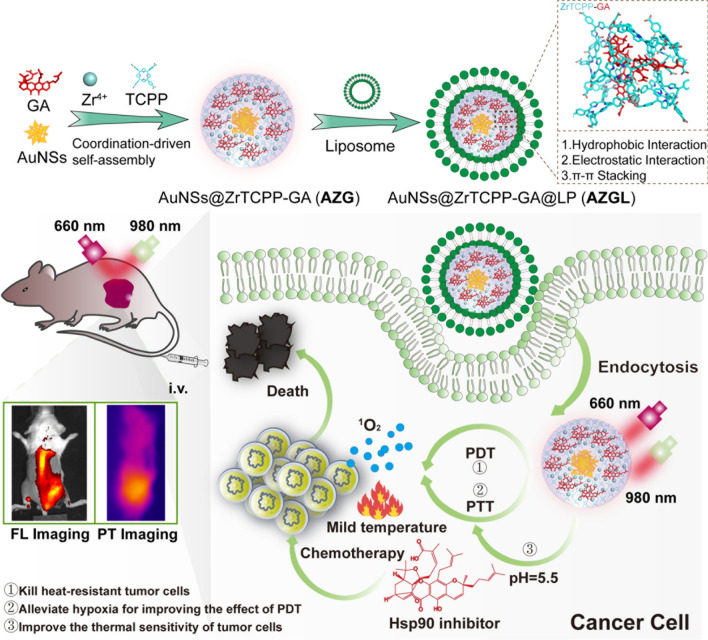
The synthetic illustration of **AZGL** nanocomposite formation and its synergistic PTT-PDT-chemotherapy for breast cancer

In this work, we demonstrate that AuNS can be used to guide the growth of the NMOF ZrTCPP, which simultaneously encapsulates GA via a facile one-pot coordination reaction. PEGylated liposome (LP) was subsequently coated on the surface of the NMOF to form the final AuNS@ZrTCPP-GA@LP (**AZGL**) nanocomposite. We found that the obtained **AZGL** platform exhibited pH-dependent dissociation behaviors and could allow sustained release of GA and TCPP moieties, which induce TCPP-mediated PDT and AuNS-mediated PTT, in synergy with the inhibitory effect on HSP90 of GA to cause cell death in vitro. The **AZGL** nanoplatform showed good accumulation ability in tumors after intravenous administration, as evidenced from the fluorescence and photothermal imaging studies. Compared with PDT or PTT alone, the combination of PDT and PTT resulted in strong synergy to exert potent antitumor efficacy against breast cancer in vivo (Scheme 1). This work highlights a facile method for the preparation of multifunctional nanoplatform toward the effective synergistic therapy of cancer.

## Methods

### Materials and measurements

Zirconyl chloride octahydrate (ZrOCl_2_·8H_2_O), tetrakis (4-carboxyphenyl) porphyrin (TCPP) and polyvinyl pyrrolidone (PVP, Mw: 10,000) were purchased from Shanghai Aladdin Biochemical Technology Co., Ltd. Gambogic acid (GA), the disodium salt of 9,10-anthracenedipropionic acid (ADPA), 2′,7′-dichlorofluorescin diacetate (DCFH-DA), methyl-thiazolyl diphenyl-tetrazolium bromide (MTT) and 4',6-diamidino-2-phenylindole (DAPI) were obtained from Zhengzhou Acme Chemical Co., Ltd. 1,2-Distearoyl-*sn*-glycero-3-phosphocholine (DSPC), cholesterol, and 2-distearoyl-*sn*-glycero-3-phosphoethanolamine-N-[carboxy(polyethylene glycol)-2000] (ammonium salt) (DSPE-PEG-COOH) were purchased from A.V.T. (Shanghai) Pharmaceutical Co., Ltd. All reagents were used as received unless specified.

LysoTracker staining kit, Calcein-AM/(propidium iodide) PI double stain kit, Annexin V/PI cell apoptosis/necrosis kit, membrane protein extraction kit, and polyvinylidene difluoride (PVDF) membrane was purchased from Beyotime Institute of Biotechnology (China). Aspartate transaminase (AST), alanine transaminase (ALT), alkaline phosphatase (AKP), and blood urea nitrogen (BUN) levels were measured with an assay kit (NanJingJianCheng, China). All reagents relevant to cell culture were obtained from GIBCO Invitrogen Corp.

The Ultraviolet–visible (UV–Vis) spectroscopy was measured on a Perkin Elmer LS55 UV–Vis spectrophotometer. Fourier-transform infrared spectroscopy (FT-IR) was implemented on a Nicolet MagNa-IR 550 infrared spectrometer (Thermo Fisher, USA) as KBr pellets. Emission spectrometry was measured on a Perkin Elmer LS55 spectro-fluorophotometer. Powder X-ray diffraction (PXRD) patterns were recorded on the X’Pert-Pro MPD diffractometer (Netherlands PANalytical). Nitrogen (N_2_) adsorption isotherms were recorded using a BELSORP-max (BEL, Japan). X-ray photoelectron spectroscopy (XPS) was measured with a Thermo Fisher Scientific Nexsa spectrometer. Inductively coupled plasma-mass spectrometry (ICP-MS) was taken on an ICAP 6300 of Thermo Scientific. The hydrodynamic diameters and zeta-potentials of samples were determined by Zetasizer Nano-ZS ZEN3600 (Malvern Instruments, UK). Transmission electron microscope (TEM) and energy-dispersive X-ray spectroscopy in TEM (TEM-EDS) were performed on JEOL JEM 100CXII operated at 200 kV. Fluorescence microscopy images were observed on a confocal laser scanning microscopy (CLSM, Nikon C1-Si TE2000, Japan). Flow cytometry was performed using a BD FACS Caliber instrument (BD Biosciences, San Jose, USA). Cytotoxicity assay was carried out on a multifunction microporous plate detector (Infinite M1000 Pro, Tecan, Switzerland). In vivo image acquisition was performed on IVIS spectrum imaging system (USA). The high-performance liquid chromatography (HPLC) was carried out on a Shimadzu HPLC system (Shimadzu, Japan).

### Preparation of AuNS, AZ/AZG and AZGL

AuNS were prepared through the seed-mediated growth method with optimization [27]. Briefly, the seed solution was prepared by adding 5 mL of 38.8 mM citrate solution to 50 mL of boiling chloroauric acid [HAuCl_4_, 0.03% *w* (g)/*v* (mL)] solution under stirring at 600 rpm. After 20 min, the solution was cooled and then kept at 4 °C for long-term storage.

Preparation of AuNS: 100 µL of the above citrate-stabilized seed solution (10 nM, 12 ± 0.7 nm) was added to the mixture of HAuCl_4_ (10 mL, 0.25 mM) and HCl solution (10 µL, 1 M) in a 50 mL glass vial under ice bath with stirring at 200 rpm. Subsequently, 100 µL silver nitrate (AgNO_3_, 3 mM) and 50 µL of ascorbic acid (AA, 100 mM) were added simultaneously and the color turned from wine-red to blue-purple after stirring for 60 s. PVP (500 µL, 10%) was added to the above solution. Immediately afterward, one centrifugal wash was performed with deionized (DI) water at 11,000 rpm for 10 min to halt the nucleation. The precipitation was re-dispersed in DI water and filtered by a 0.22 μm microporous membrane to afford the AuNS solution, and then kept at 4 °C for long-term storage.

Preparation of **AZG**: The above 1 mL AuNS (300 µg/mL) solution and 2 µL GA (5 mg/mL) solution were mixed with TCPP and ZrOCl_2_·8H_2_O in equivalent molar ratio. After stirring at room temperature for 30 min, the core–shell composite of **AZG** was collected by centrifugation (5000 rpm, 7 min), washed three times with DI water, and re-dispersed in 1 mL of DI water. The GA drug encapsulation efficiency (EE% = drug encapsulated/original input × 100%) and drug loading capacity [DL% = drug encapsulated/(drug + all materials input) × 100%] were determined by HPLC with detection wavelength of 361 nm. The synthetic process for **AZ** was similar to that of **AZG** but in the absence of GA, and the ZrTCPP NMOF was synthesized following the protocol for **AZ** but in the absence of AuNS.

Preparation of liposome coated **AZG** (**AZGL**): 24 µL of 0.1 M DSPC, 24 µL of 1.0 mM DSPE-PEG-COOH, and 15 µL of 50 mM cholesterol were added into the flask with 3 mL of chloroform [28], which was mixed uniformly at 40 °C. Chloroform was then volatilized under reduced pressure and dried under argon. Afterward, 2 mL of **AZG** (1 mg/mL) was added into the flask, followed by shaking at 30ºC to dissolve the thin film. The final solution was extruded through a 0.2 µm polycarbonate membrane filter back and forth fifteen times, and the formed liposome**-AZG** (**AZGL**) composite was washed with DI water and collected by centrifugation (10,000 rpm, 7 min), and kept at 4ºC for further use.

### Super-resolution imaging of AZGL

The glass culture dish was first activated by poly-L-lysine (1 mg/mL) solution for 30 min, followed by careful washing to remove the excess poly-L-lysine. Then the **AZGL** (50 μg/mL) was added for 1 h fixation. Excessive **AZGL** has washed away and membrane-stained fluorescent dye PKH67 was employed to label the liposome (LP) on the **AZGL**. The prepared samples were observed under super-resolution microscopy. Ex_PKH67_: 488 nm (green); Ex_**AZGL**_: 561 nm (red).

### In vitro release of GA and TCPP

The pH-responsive release profiles of GA and TCPP from **AZGL** nanocomposite were obtained using a widely adopted procedure [29]. Briefly, **AZGL** nanoparticles (10 mg) were dispersed in 10 mL of HEPES (pH 5.5 or 7.4) and then incubated at 37 °C. At different time points, a 0.5 mL aliquot was withdrawn from each sample and centrifuged to collect the supernatant. The cumulative amounts of TCPP released from **AZGL** were determined according to the UV–Vis absorption at 410 nm with the standard calibration curve. Meanwhile, the cumulative amounts of GA were determined according to the UV–Vis absorption intensity at 361 nm by HPLC.

### Theoretical simulation

To determine the coordination environment of Zr^4+^ with TCPP ligand, density functional theory (DFT) calculation was carried out using the Gaussian09 package. Full geometry optimizations of TCPP, Zr(TCPP), and Zr(TCPP)_2_ compounds with different structures in the water phase were carried out using Becke’s three-parameter hybrid (B3LYP) exchange–correlation functional. The standard split-valence 6-31G(d) basis set of atomic orbitals was used for the C, H, N, and O atoms, and Zr^4+^ was described by the Stuttgart/Dresden (SDD) relativistic pseudopotentials. For geometrically equilibrated complexes, the energies of highest occupied molecular orbital (E_HOMO_) and lowest unoccupied molecular orbital (E_LUMO_) were obtained. The energy gap (ΔE) was then calculated by the following equations:

1 $$\Delta {\text{E}} = {\text{E}}_{{{\text{LUMO}}}} - {\text{E}}_{{{\text{HOMO}}}}$$

The interactions between the compounds of TCPP, Zr(TCPP), Zr(TCPP)_2,_ and Zr(TCPP)_2_-GA with gold surface were optimized and the energy was calculated using the Forcite module of Materials Studio 7.0 software in the COMPASS force field. The values of adsorption energy (E_ads_) between compounds and Au(111) surface were calculated as follows [30]:

2 $${\text{E}}_{{{\text{ads}}}} = {\text{E}}_{{{\text{total}}}} - \left( {{\text{E}}_{{{\text{surface}}}} + {\text{E}}_{{{\text{compound}}}} } \right)$$

where E_total_ presents the total energy of the simulation system, E_surface_ presents the energy of Au(111) surface and E_compound_ presents the energy of TCPP, Zr(TCPP), Zr(TCPP)_2_ or Zr(TCPP)_2_-GA.

### Photothermal efficiency determination

To evaluate the photothermal conversion efficiency of **AZGL**, 200 µL of **AZGL** aqueous solutions (12.5, 25.0, 50.0 µg/mL) were exposed to a 980 nm laser irradiation at different power densities (0.5, 1.0, and 1.5 W/cm^2^) for 10 min. Subsequently, the solutions were allowed to cool down naturally for 10 min. The temperature changes were recorded by an infrared thermal imaging camera every 30 s. The photothermal conversion efficiency (*η*) can be calculated according to the Eq. (3) [31]

3 $$\eta = \frac{{{\text{hs}}(T_{max} - T_{surr}) - Q_{dis}}}{{I(1 - 10_{{}}^{{ - {\text{A}}980}} )}}$$

wherein the T_max_ (K) means the equilibrium temperature, T_surr_ (K) is the ambient temperature of the surroundings. The Q_dis_ is heat loss from light absorbed by the container, and it is calculated to be approximately equal to 0 mW. I (W) represents incident laser power (0.5/1.0/1.5 W/cm^2^), A_980_ is the absorbance of samples at 980 nm. The h (W·cm^−2^ K^−1^) means heat transfer coefficient and s (cm^2^) represents the surface area of the container. The hs is calculated using the following Eq. (4)

4 $$\tau _{\text{s}} = \frac{{{\text{m}_{D}\text{c}_{D}}}}{hs}$$

wherein τ_s_ is the sample system time constant, m_D_ and c_D_ are the mass (0.2 g) and heat capacity (4.2 J/g) of the solvent.

### Detection of singlet oxygen in vitro

The generation of singlet oxygen (^1^O_2_) was detected chemically using the ADPA probe [32], which converts to its nonfluorescent endoperoxide in the presence of ^1^O_2_. Separate portions of 20 µL of ADPA (7.5 mM) were respectively mixed with 2 mL of AuNS (36 µg/mL), ZrTCPP (8 µg/mL) or **AZG** (45 µg/mL) which contains AuNS (36 µg/mL) and ZrTCPP (8 µg/mL). The samples were irradiated with a 660 nm laser (0.22 W/cm^2^), and the optical density (OD) values at 378 nm were recorded by a UV–Vis spectrophotometer.

### Anti-immune clearance of AZGL

To evaluate the anti-immune clearance of **AZGL** and **AZG**, RAW264.7 cells were seeded and further incubated in 35-mm cell culture dishes with 3 × 10^5^ cells/dish for 24 h, followed by adding **AZGL** (60 µg/mL) or **AZG** (45 µg/mL) as control and incubated for another 12 h. The cells were washed with PBS three times and analyzed by CLSM (E_x_ = 640 nm) and flow cytometry.

### Subcellular location of AZGL

4T1 cells were seeded and further incubated in 35-mm cell culture dishes with 3 × 10^5^ cells/dish for 24 h, and then incubated with **AZGL** (60 µg/mL) for another 9 h. The cells were washed with PBS and stained with LysoTracker staining kit (500 nM) for 30 min, which was then washed with PBS three times before for CLSM (E_x_ = 640 nm).

### Cellular uptake behaviors of AZG and AZGL

4T1 cells were seeded in 35-mm cell culture dishes with 3 × 10^5^ cells/dish and incubated for 24 h. Upon removal of the culture, **AZGL** (60 µg/mL) and **AZG** (45 µg/mL, as control) were added into the well for 6 h, 9 h, 12 h. The fluorescence intensity of cells was determined by CLSM (E_x_ = 640 nm) to investigate the correlation between the uptake of **AZGL** and **AZG** with the incubation time.

### Intracellular reactive oxygen species (ROS) assay

The intracellular ROS level was measured via CLSM using DCFH-DA as the ROS indicator. 4T1 cells were seeded and further incubated in the 24-well plates with 1 × 10^5^ cells/well for 24 h. After treating the cells with **AZGL** (60 µg/mL) for 9 h, the medium was removed, DCFH-DA was then added with a final concentration being 4 µM and incubated for 30 min. Thereafter, the cells were further irradiated with a 660 nm laser (0.22 W/cm^2^) for 5 min. Then all the cells were viewed by CLSM (Ex = 488 nm).

### MTT assays

The in vitro cytotoxicity to MDA-MB-231 and 4T1 cells was determined by MTT assay. The cells were seeded into the 96-well plates with 5 × 10^3^ cells/well and incubated at 37 °C for 24 h. Then the original medium was replaced with another 100 µL of fresh medium containing different concentrations of **AZL** or **AZGL** (0, 15, 30, 45, and 60 µg/mL). After incubation for 9 h, the medium was replaced by 100 *µ*L of fresh medium. For the control groups, the cells were cultured in dark all the time. For the PDT group, the cells were irradiated with a 660 nm laser (0.22 W/cm^2^, 5 min). For the PTT groups, the cells were irradiated with a 980 nm laser (0.5 W/cm^2^, 10 min). For combined therapy, the cells were irradiated with a 980 nm laser (0.5 W/cm^2^, 10 min) and subsequently irradiated with a 660 nm laser (0.22 W/cm^2^) for 5 min. Then the cells were cultured for another 24 h and 20 µL of MTT (5 mg/mL in PBS) added. The cells were further incubated for 4 h. After that, the supernatants were carefully removed, and 100 µL of DMSO was added to each well. After shaking for several minutes, the OD values were recorded at 490/570 nm on a microplate reader (Infinite M1000 Pro, Tecan, Switzerland). The relative cell viability was calculated by the following equation: cell survival rate (%) = [(A_s_ − A_b_)/(A_c_ − A_b_)] × 100%, in which A_s_ is the absorbance in the presence of samples, A_c_ is the absorbance in the absence of samples, and A_b_ is the absorbance of the blank.

### Western blot analysis

To study the influence of GA on HSP90 expression, 4T1 cells were seeded in a 6-well plate with 4 × 10^5^ cells/well and incubated for 24 h. Then, the culture medium was removed, **AZL** and **AZGL** (60 µg/mL) were added for another 9 h incubation. Then the **AZL** and **AZGL** groups were irradiated with/without 980 nm laser (0.5 W/cm^2^, 10 min). The cells were lysed and the protein was harvested on the ice. Afterward, the samples were centrifuged at 12,000 rpm for 10 min and the supernatants were collected for protein determination by a microplate reader. The protein with 20 µg/well was loaded on SDS–polyacrylamide gels for electrophoresis. Samples were separated on electrophoresis and the blots were transferred to PVDF membrane. Skimmed milk was used to block a protein in TBST (Tris-buffered saline with 0.05% Tween-20, pH 7.4) for 30 min, and then the primary antibody was incubated with membranes at 4 °C overnight. Finally, the membranes were rinsed with TBST for three times, and further cultured with anti-rabbit horseradish peroxidase (HRP)-conjugated secondary antibodies for 12 h. Protein bands of HSP90 and glyceraldehyde-3-phosphate dehydrogenase (GAPDH) were recorded by Image Lab camera (Fluor Chem R, USA).

### Living/dead cell staining experiment

4T1 cells were seeded in 6-well plates with 3 × 10^5^ cells/well and incubated for 24 h and then treated with **AZGL** (60 µg/mL). After further incubation for 9 h, the medium was replaced with a fresh medium. For PTT, the cells were irradiated with a 980 nm laser (0.5 W/cm^2^, 10 min). For PDT, the cells were irradiated with a 660 nm laser (0.22 W/cm^2^, 5 min). For combined therapy, the cells were irradiated with a 980 nm laser (0.5 W/cm^2^, 10 min) and subsequently irradiated with a 660 nm laser (0.22 W/cm^2^, 5 min). The cells were stained with Calcein-AM (8 µM) and PI solutions (2 µM) in PBS buffer solution and incubated for 30 min. The cells were washed with PBS buffer solution and viewed by CLSM. The Calcein-AM and PI were excited with lasers at 488 and 543 nm, respectively.

### Annexin V/propidium iodide (PI) staining experiment

The type of cell death analysis for 4T1 cells was performed by utilizing a flow cytometric assay of Annexin V and PI co-staining. 4T1 cells were seeded in 24-well plates with 1 × 10^5^ cells/well and incubated for 24 h and then treated with **AZGL** (60 µg/mL). After co-incubation for 9 h, the medium was replaced with a fresh medium. For PTT, the cells were irradiated with a 980 nm laser (0.5 W/cm^2^, 10 min). For PDT, the cells were irradiated with a 660 nm laser (0.22 W/cm^2^, 5 min). For combined therapy, the cells were irradiated with a 980 nm laser (0.5 W/cm^2^, 10 min) and subsequently irradiated with a 660 nm laser (0.22 W/cm^2^, 5 min). Then, 4T1 cells were stained with Annexin V/PI for 20 min. Subsequently, the cells were collected for flow cytometric measurement.

### Hemolysis tests

The hemocompatibility of **AZGL** were determined according to the established standard (ISO10993-4). Briefly, the fresh mice blood was obtained from the animal experimental center of Southern Medical University and used in compliance with a local ethics committee (Permit Number: 44002100030137). Subsequently, red blood cells (RBCs) were isolated from plasma after careful washing. The suspension of RBCs at a final concentration of 2% (*v*/*v*) was added to **AZGL** solution with varying concentrations from 10 to 200 µg/mL and then incubated at 37 °C in a thermostatic water bath for 3 h. PBS and Triton X-100 (10 g/mL, a surfactant known to lyse RBCs) were used as negative and positive controls, respectively. After centrifugation (12,000 rpm, 15 min), 200 μL of the supernatant of each sample was transferred to a 96-well plate. The free hemoglobin in the supernatant was monitored through a microplate reader at 540 nm. The hemolysis ratio of RBCs was measured with Eq. (5).

5 $${\text{Hemolytic ratio (\% ) }} = \, \frac{{{{A}_\text{sample}} - A_{\text{negative control}}}}{{A_{\text{positive control}} - A_{\text{negative control}}}} \times 100\%$$

where *A*_sample_, *A*_negative control_ and *A*_positive control_ were denoted as the absorbance of sample, negative and positive controls, respectively.

### In vivo fluorescence and photothermal imaging

For fluorescence imaging: BALB/c female mice of 4−6 weeks were subcutaneously inoculated 4T1 cells (2 × 10^6^ cells, diluted in 100 µL PBS) at the right-back of the hind leg region. When the tumors grew to 150 mm^3^ in volume, 200 µL of **AZGL** solution (1 mg/mL) were injected into the mice by the intravenous method. Thereafter, the mice were anesthetized and imaged by an IVIS Lumina III Imaging System (Caliper, USA) at 0, 3, 6, 12, 18, 24, 36, and 48 h post-injection. The excitation wavelength was 640 nm and the fluorescence emission at 670 nm was collected. For the tissue distribution study of the composites, the mice were sacrificed at 24 h post-injection, and major organs were collected for ex vivo imaging. To quantitatively assess the biodistribution of the **AZGL**, major organs (heart, liver, spleen, lung, and kidney) and the tumor tissues were digested to determine the amounts of Au using ICP-MS at 24 h post-injection.

For photothermal imaging: When the tumors reached an approximate size of 150 mm^3^ in volume, 200 µL of **AZGL** solution (1 mg/mL) were injected into the mice by the intravenous method. The mice of the control group were injected with PBS buffer. After 24 h, infrared thermal imaging was measured by an infrared camera (Fluke TiS60, USA) under irradiation with a 980 nm laser at a power density of 0.5 W/cm^2^ and the temperature of mice was recorded by Smart View 3.15 software.

### In vivo photothermal/photodynamic synergistic therapy

When the tumor reached about 150 mm^3^, the mice were randomly divided into 7 groups (n = 5), i.e., (a) control group (PBS injection), (b) PBS with 660 nm light for 5 min and 980 nm laser irradiation for 10 min (PBS + PDT + PTT), (c) GA, (d) **AZGL** (No light), (e) **AZGL** with 660 nm laser irradiation for 5 min (**AZGL** + PDT), (f) **AZGL** with 980 nm laser irradiation for 10 min (**AZGL** + PTT) and (g) **AZGL** with 660 nm light for 5 min and 980 nm laser irradiation for 10 min (**AZGL** + PDT + PTT). The mice were intravenously injected with samples of **AZGL**. After that, the weight and tumor volume of mice were recorded every day. The tumor volume was calculated by the formula of V = (a × b^2^)/2, in which a and b represent the length and width of the tumor. After 14 days, the mice were sacrificed, the main organs and tumors were collected, and the tumors were photographed. Then the organs and tumors were sliced and stained with Hematoxylin and Eosin (H&E), and the histopathological examination was carried out under the microscope.

### Application safety evaluation

For blood biochemical parameter analysis, the mice were administered intravenously with drugs after different treatments. 14 days later, the blood sample of mice was collected and centrifuged at 3500 rpm for 10 min to obtain plasma samples for measuring the clinical parameters by an assay kit, including liver-related alkaline phosphatase (AKP), alanine aminotransferase (ALT), aspartate aminotransferase (AST), and kidney-associated blood urea nitrogen (BUN).

### Hypoxia-inducible factor-1α (HIF-1α) immunostaining

4T1 tumor-bearing mice (n = 3) were randomly assigned to four groups and treated with (a) **AZGL** (No light), (b) **AZGL** (PDT), (c) **AZGL** (PTT), (d) **AZGL** (PDT + PTT). Briefly, 200 µL of **AZGL** solution (1 mg/mL) were injected into the mice by the intravenous method, 14 days post-treatment, the collected tumor tissues were fixed and sliced. The tumors were excised and fixed in 4% formalin for HIF-1α staining. The slices were stained with rabbit anti-HIF-1α (1:1000, Abcam) monoclonal antibody overnight at 4 °C. Subsequently, the secondary antibodies conjugated with FITC were incubated with slices for 1 h. After staining with DAPI, the immunofluorescence staining images were captured using CLSM.

### Statistical analysis

Differences among samples were calculated with the two-tailed Student’s t-test using an independent samples t-test in SPSS 16.0. Differences among groups were considered statistically significant at ***p < 0.001, **p < 0.01, and *p < 0.05.

## Results and discussion

### Preparation and characterization of AZGL

We firstly optimized the self-assembly conditions of ZrTCPP by fixing the concentration of AuNS at 300 μg/mL while increasing the loading of Zr^4**+**^ and TCPP with an equivalent molar ratio. The UV–Vis spectrum showed that the absorption at 410 nm of the formed AuNS@ZrTCPP (**AZ**) reached the maximum when both concentrations of Zr^4**+**^ and TCPP were 0.15 mM (Additional file 1: Figure S1A, B). To avoid the formation of AgCl nanoparticles during the reduction of Au^3+^, AgNO_3_ coupled with ascorbic acid was added into the reaction solution, the ascorbic acid could reduce Ag^+^ to the Ag element immediately before the formation of AgCl [33]. During the synthetic process of AuNS, the product will be formed as polydisperse rods and spheres with low photothermal conversion efficiency in the absence of Ag^+^ [27]. Ag^+^ is thought to have a key role in the anisotropic growth of Au branches on certain crystallographic facets on multi-twinned citrate seeds, rather than in the formation of Ag branches which is well discussed in the literatures [27, 34, 35]. The PXRD peaks of AuNS was observed (Additional file 1: Figure S3A): 38.3°, 44.4°, 64.7°, 77.7°, and 81.8° are indexed to (111), (200), (220), (311), and (222) planes of Au with face-centered cubic structure, respectively, which is consistent with the results of the literature [36, 37]. While the PXRD peaks of AgCl, indexed as (111), (200), (220), (311), (222), (400) and (420) planes, and respectively positioned at 27.8°, 32.3°, 46.3°, 54.7°, 57.5°, 67.4° and 76.1°, were not identified in our case [38]. We, therefore, conclude that there is no AgCl nanoparticles introduced in our case. In order to find out where the Ag nanoparticles potentially go, we further conducted the energy dispersive X-ray spectroscopy (EDS) for the AuNS. The results indicate that the mass content of Ag nanoparticles in AuNS is around 11.96% (Additional file 1: Figure S2), which may has a certain contribution to the photothermal conversion efficiency of AuNS [33]. Powder X-ray diffraction (PXRD) patterns demonstrated that ZrTCPP has only a broad absorption peak at 3°–30°. For **AZ**, it exhibits a good match for the diffraction peaks between the ZrTCPP and AuNS (Additional file 1: Figure S3A), indicating its successful synthesis. The N_2_ adsorption–desorption analysis revealed that the maximum N_2_ uptake of **AZ** was only 4 cm^3^/g, suggesting the nonporous nature of **AZ** (Additional file 1: Figure S3B), probably related to the amorphous nature of the NMOF which was synthesized in the water [39]. To effectively load GA, we mixed it with AuNS before the formation of NMOF ZrTCPP, presuming that carboxyl and phenolic groups in GA may promote its interaction to the surface of AuNS and NMOF ZrTCPP. Thus, assembly of Zr^4+^ with ligand TCPP in the presence of prepared AuNS and GA in one-pot afforded AuNS@ZrTCPP-GA (**AZG**) nanocomposite.

It was reported that the IC_50_ value of GA against breast cancer 4T1 cells is 0.12 µg/mL [40]. We further verified the safety of GA on RAW 264.7 macrophages, and the IC_50_ value was measured to be 2.09 μg/mL (Additional file 1: Figure S4). Concerning the high toxicity of GA, the encapsulation rate and loading rate of GA were respectively controlled to be 38.40 ± 0.57% and 1.04 ± 0.01% by HPLC (Additional file 1: Figure S5A, B). In the final step, LP was applied to coat **AZG** to form AuNS@ZrTCPP-GA@LP (**AZGL**) in an attempt to reduce the recognition by the host immune system to prolong the circulation time in the blood and to improve the accumulation in tumors via the enhanced permeability and retention (EPR) effect. In **AZGL**, the contents of TCPP and AuNS were calculated by the UV–Vis spectra (Additional file 1: Figure S5C, D) to be 12.58 ± 1.31% and 60.14 ± 0.62%, respectively. The content of Zr^**4+**^ was 0.84 ± 0.03% through the ICP-MS and the liposome was about 25.82%. The molar ratio of TCPP to Zr^**4+**^ is about 2:1 according to the above calculations.

**Fig. 1:**
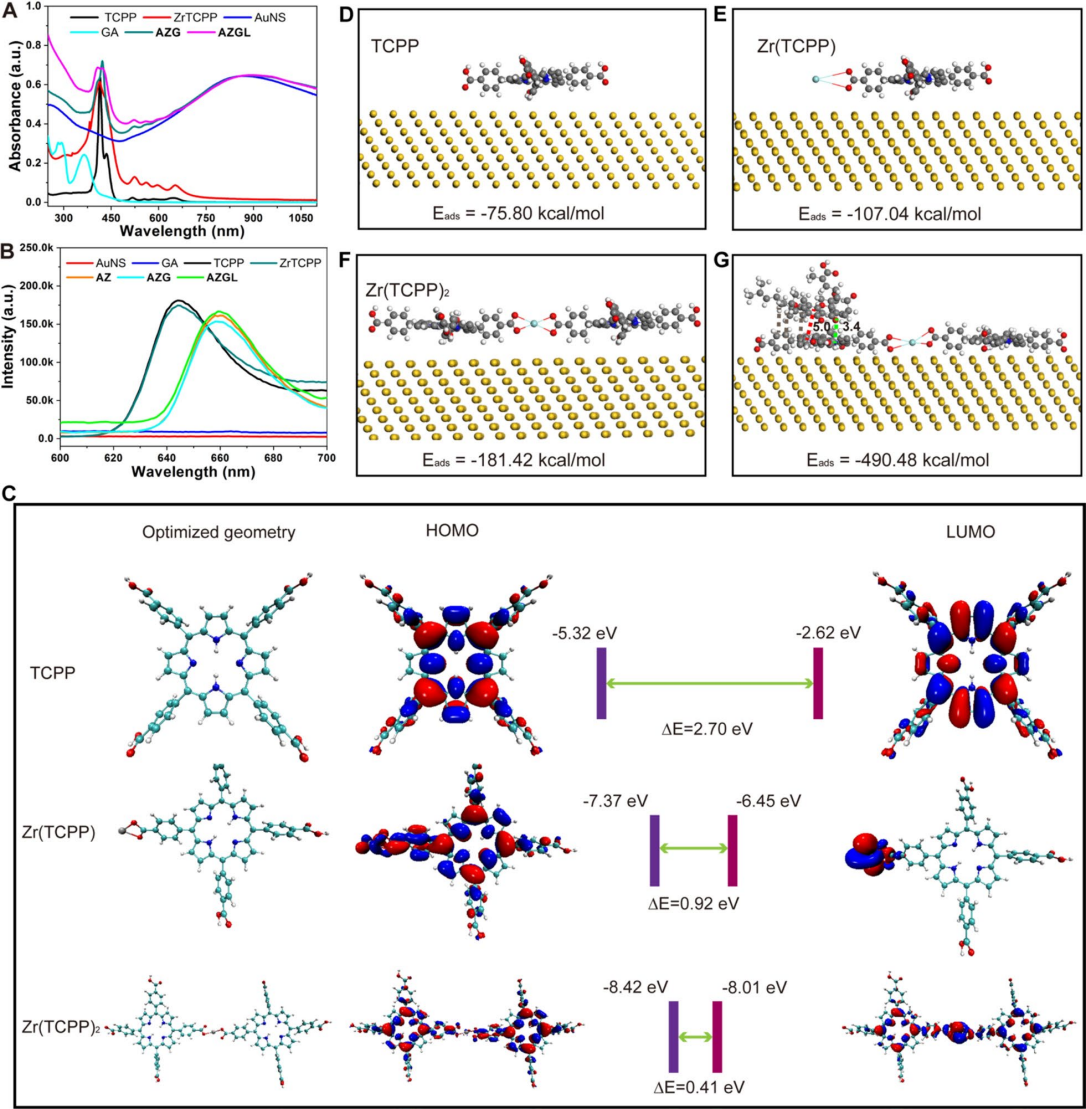
**A** UV–Vis spectrum of TCPP, ZrTCPP, GA, AuNS, **AZG**, and **AZGL**. **B** The fluorescence spectrum (E_x_ = 410 nm) of TCPP, ZrTCPP, GA, AuNS, **AZ**, **AZG**, and **AZGL**. **C** The optimized structures and HOMO–LUMO distributions of TCPP and Zr(TCPP) and Zr(TCPP)_2_ were calculated by DFT (Gaussian 09/B3LYP/6-31G). Side views of configurations of TCPP (**D**), Zr(TCPP) (**E**), Zr(TCPP)_2_ (**F**), and Zr(TCPP)_2_-GA (**G**) on Au(111) surface obtained from geometry optimization, the gray dotted line indicated hydrophobic interaction, the green solid line indicated hydrogen bonding interaction, and the yellow dotted line indicated π–π stacking

In the FT-IR spectrum of free TCPP (Additional file 1: Figure S6), the absorption peak at 1690 cm^−1^ is assignable as the stretching vibration of C=O. After the formation of **AZ** and **AZG**, this peak is blue-shifted to 1658 cm^−1^, which proved that the C=O coordinated with Zr^**4+**^. In addition, the absorption peak of the **AZ** and **AZG** at 963 cm^−1^ (assignable as the N−H vibration) did not shift significantly in comparison to TCPP, suggesting that Zr^**4+**^ did not coordinate with the nitrogen atoms of the porphyrin skeleton [41]. Similarly, the UV–Vis spectrum (Fig. 1A) showed that the Soret band (B band) of **AZG** was broader than that of the free TCPP ligand due to its relatively stronger aggregation as compared to free TCPP under UV–Vis conditions [42]. Meanwhile, the four weak absorption Q bands in the 500–750 nm remain unchanged, indicating that there is no chelation of the Zr^4+^ by the four N atoms for the porphyrin skeleton [43].The fluorescence spectra show that both free TCPP ligand and ZrTCPP NMOF exhibit similarly strong fluorescence emission in the range of 640−660 nm under the excitation of 410 nm (Figure 1B). Nevertheless, the fluorescence was red-shifted after the formation of **AZG** and **AZGL**. The possible reason for the red-shift of the fluorescence of TCPP could be the fluorescence resonance energy transfer (FRET) between TCPP and AuNS [44] because the fluorescence of **AZ** also underwent the same degree of red-shift. The red fluoresce signal provided an opportunity for **AZGL** to be used as a general diagnostic platform.

As discussed above, the molar ratio of Zr^4+^ and TCPP was about 1:2 and the Zr^4+^ and TCPP were coordinated through the carboxylate group. To gain a deeper insight into the self-assembly mechanism of ZrTCPP [30], DFT-based quantum chemical calculations were used to clarify the optimized structure, spatial distributions of HOMO and LUMO of TCPP, Zr(TCPP), and Zr(TCPP)_2_. As shown in Fig. 1C, the electron density distributions in the HOMO of TCPP, Zr(TCPP), and Zr(TCPP)_2_ were mainly located on pyridines, which implied the possible migration of electrons from the pyridines to the empty orbitals. Meanwhile the electron density distributions in the LUMO of Zr(TCPP) and Zr(TCPP)_2_ were mainly distributed on Zr and its adjacent carboxylate group. This distribution may be favorable for the adsorption on the AuNS surface through the acceptance of electrons being responsible for d orbitals of metal atoms. The calculated ΔE values for TCPP, Zr(TCPP), and Zr(TCPP)_2_ are 2.70, 0.92, and 0.41 eV respectively, which means that the global reactivity of TCPP, Zr(TCPP), and Zr(TCPP)_2_ molecules is increased in order. These results proved that Zr(TCPP)_2_ molecule has the strongest adsorption on the AuNS surface, and the ZrTCPP shell is assembled on the AuNS surface by the formation of Zr(TCPP)_2_ NMOF. Subsequently, the adsorption behaviors of TCPP, Zr(TCPP), and Zr(TCPP)_2_ on Au(111) surface with minimum surface energy [45] were optimized and the energy was calculated using the Forcite module in the COMPASS force field (Fig. 1D–F). The adsorption energies of TCPP, Zr(TCPP), and Zr(TCPP)_2_ on Au (111) surface were computed as − 75.80, − 107.04 and − 181.42 kcal/mol respectively, indicating the interaction between Zr(TCPP)_2_ molecule and Au(111) surface was stronger than the other molecules. Therefore, the result is highly consistent with the distributions of HOMO and LUMO. To explain the interaction between GA and **AZ**, we placed GA on the surface of **AZ** for structural optimization and energy calculation. As shown in the Fig. 1G, the hydroxyl group of GA formed a hydrogen bond (3.4 Å) with the nitrogen on the pyridine ring of TCPP, and the benzene ring of GA formed a π–π stacking (5.0 Å) with the pyridine ring of TCPP. In addition, multiple hydrophobic bonds were formed between GA and Zr(TCPP)_2_. These forces provided strong adsorption energy being − 490.48 kcal/mol. Overall, these results indicated that GA can self-assemble with NMOFs ZrTCPP on the surface of AuNS through hydrophobic interaction, electrostatic interaction, and π–π stacking.

**Fig. 2:**
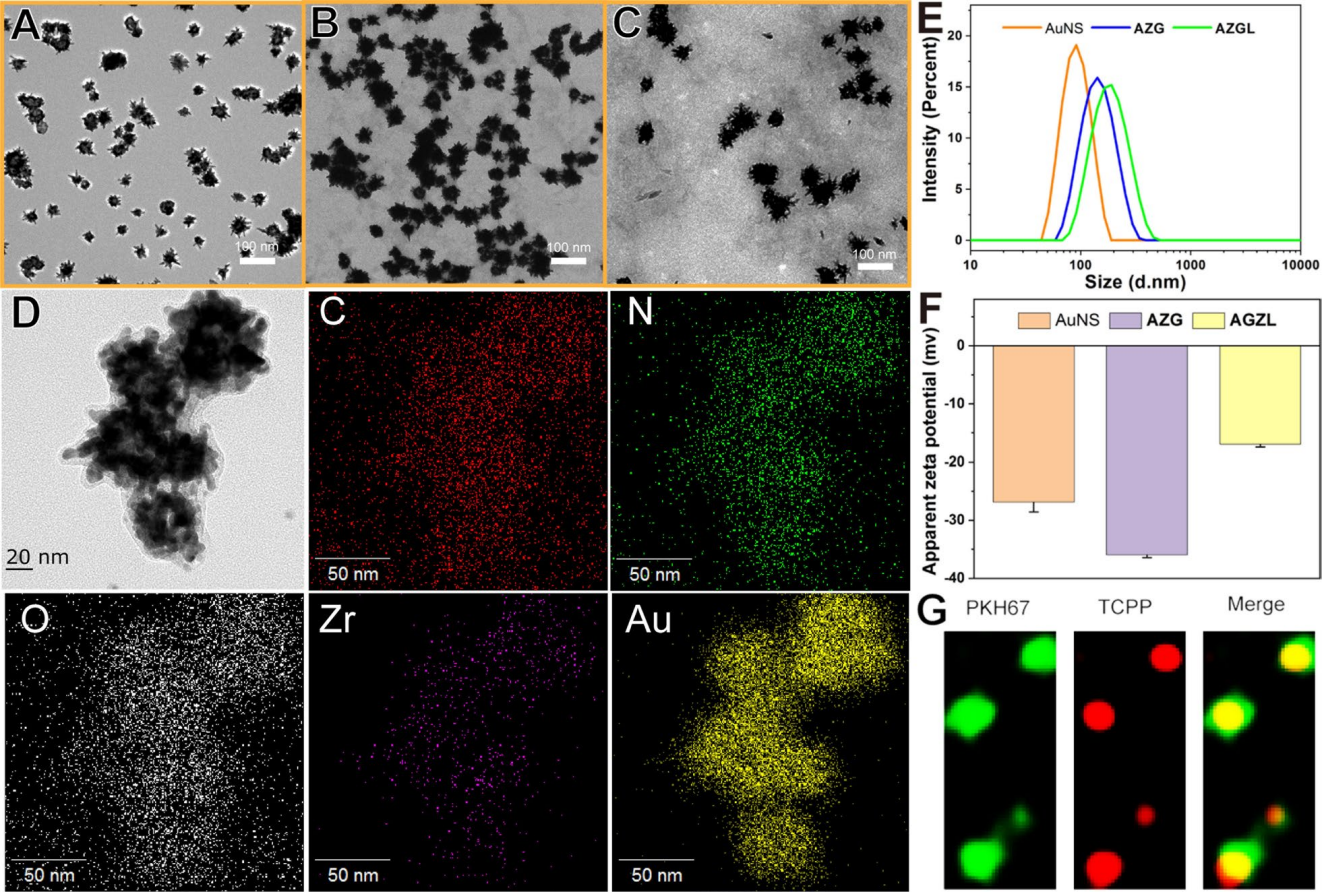
TEM images of **A** AuNS, **B** **AZG** and **C** **AZGL**. **D** TEM-EDS for **AZG**. **E** The size distribution and **F** zeta potential analysis of AuNS, **AZG**, and **AZGL**. **G** The super-resolution images of **AZGL** stained with membrane dye PKH67 (green). Ex_PKH67_: 488 nm (green); Ex_**AZGL**_: 561 nm (red)

The diameters of the previously reported AuNS are usually about 100–200 nm [46]. Although the larger size exhibits shorter blood residence time and longer biological half-life in vivo, it shows poor tissue penetration and low cellular uptake [47]. It was reported that particles about 50–100 nm have the advantages of higher tumor tissue penetration, higher cancer cell uptake, and slower immune clearance [48–50]. With these in mind, we optimized the particle size of gold seeds to be *ca.* 12 nm and prepared AuNS with the size of about 50 nm as shown in Fig. 2A. The mean diameter of the particles increased from 52.2 ± 9.2 nm of AuNS to 71.9 ± 9.1 nm of **AZG** (Fig. 2B) and 87.7 ± 9.2 nm of **AZGL** (Fig. 2C). Therefore, the constructed nanocomposite **AZGL** is compatible with the above-mentioned size requirement. In addition, the AuNS as a core (Fig. 2A) in **AZG** has obviously coated an outer layer of ZrTCPP as a shell (Fig. 2B), and the thorn-like structure of AuNS became blunt in **AZGL** (Fig. 2C). As shown in the transmission electron microscopy (TEM) mapping results (Fig. 2D), the Au, Zr, C, N, and O elements displayed homogenous distribution, which proved the formation of the composite **AZG** and showed an excellent agreement with the theoretical calculation. Similarly, in the X-ray photoelectron spectroscopy (XPS) of **AZG** (Additional file 1: Figure S7A), the binding energy assignable as Zr 3d appeared at 182.4 eV and 184.8 eV (Additional file 1: Figure S7B) and N1s at 399.5 eV (Additional file 1: Figure S7C), confirming the presence of Zr^4+^ ions and TCPP ligands. Subsequently, the hydrated particle diameter and surface charge of the nanocomposites were measured by dynamic light scattering (DLS). As shown in Fig. 1E, the mean DLS particle sizes of AuNS, **AZG**, and **AZGL** were approximate 96.5 nm (PDI 0.21), 141.8 nm (PDI 0.35), and 190.1 nm (PDI 0.24), respectively. According to the PDI value, the LP could significantly improve the aqueous dispersion of the nanocomposite. The surface charge of AuNS, **AZG**, and **AZGL** was − 26.8 mV, − 35.9 mV, and − 16.9 mV, respectively (Fig. 2F), which implied that the positively charged LP could be coated on the surface of negatively charged **AZG**. In **AZGL**, TCPP has red fluorescence, while LP with a bilayer structure could be labeled with the fluorescent dye PKH67. As shown in Fig. 2G, LP and TCPP had a good co-localization effect, which further verified that the LP was successfully coated on the surface of **AZG**. The above observations all indicated the successful construction of **AZG** and **AZGL**.

### Release of GA and TCPP from AZGL

**Fig. 3:**
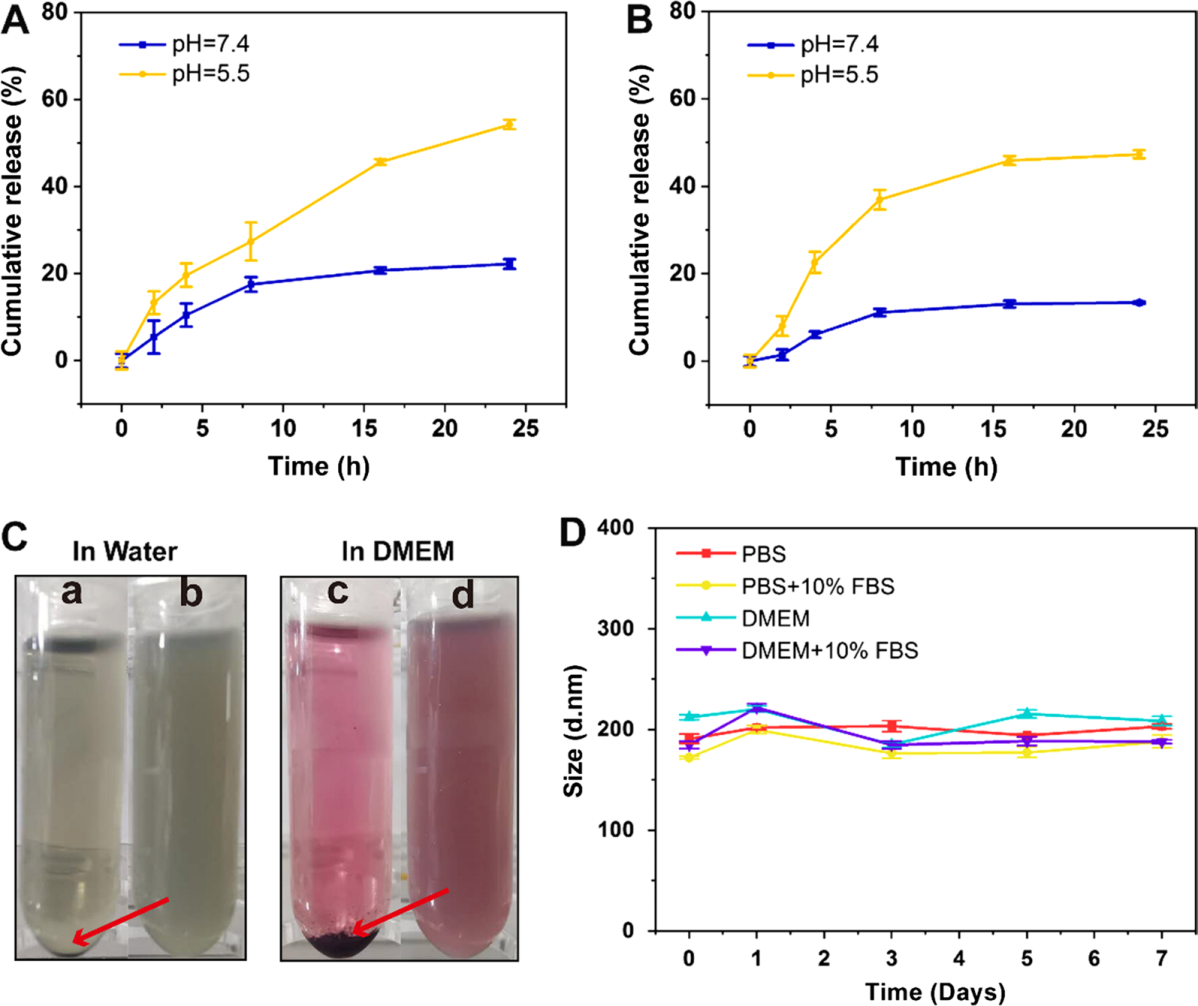
**A** The release of GA from **AZGL** at pH 5.0 and 7.4. **B** The release of TCPP from **AZGL** at pH 5.0 and 7.4. **C** The photographs of **AZG** (**a** and **c**) and **AZGL** (**b** and **d**) in water and DMEM for 24 h. **D** The long-term stability of size distribution for **AZGL** in PBS or PBS + 10% FBS and DMEM or DMEM + 10% FBS

To mimic the release behaviors of drugs in the tumor microenvironment and physiological environment, the release of GA from **AZGL** was investigated at pH 5.5 and 7.4. As shown in Fig. 3A, the release rate of GA reached 54.2 ± 1.1% in 24 h when the pH was 5.5. In contrast, it only reached 22.2 ± 1.1% when the pH was converted to 7.4. The same situation also applied to the release of TCPP (Fig. 3B). The release rate reached 47.3 ± 0.9% in 24 h at pH 5.5, but only 13.4 ± 0.2% at pH 7.4. It can be expected that the tumor acidic microenvironment would enhance the degradation of ZrTCPP-GA within **AZGL** to enable the release of loaded GA.

### In vitro evaluation of the stability of AZG and AZGL

It is important to note that the uncontrollable aggregation could occur for nanotherapeutic agents during the in vivo circulation process in the complicated biological environments, thus reducing their accumulation at the tumor site and decreasing the therapeutic efficacy [51]. Therefore, the dispersion and stability of **AZG** and **AZGL** in water and Dulbecco’s Modified Eagle Medium (DMEM) for 24 h were evaluated by DLS. As shown in Fig. 3C, both solutions of **AZG** showed obvious precipitation, while both solutions of **AZGL** showed good dispersion and stability. Further, immersing **AZGL** in PBS, PBS + 10% FBS, DMEM, and DMEM + 10% FBS solutions lead to no obvious changes in hydrodynamic diameter after seven days (Fig. 3D), indicating that **AZGL** is stable under normal physiological conditions. All these results proved that **AZGL** has remarkable physiological stability after being coated with liposome, providing strong support for the subsequent cell and animal experiments.

### In vitro PTT properties of AZGL

**Fig. 4:**
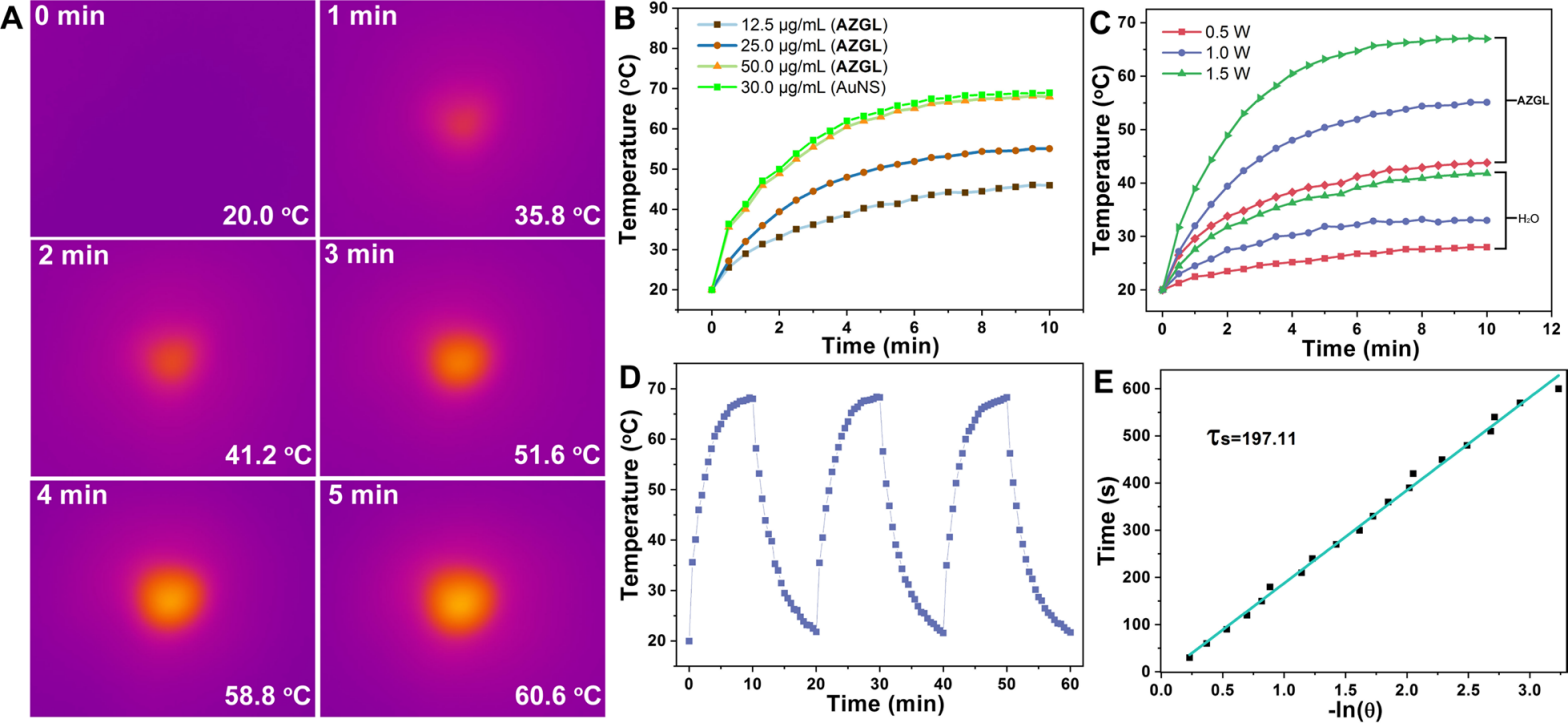
**A** The thermographic images of **AZGL** (50 μg/mL) at different times under 1.0 W/cm^2^ 980 nm laser irradiation. **B** The photothermal performance of the **AZGL** with different concentrations irradiated for 10 min by 1.0 W/cm^2^ 980 nm laser and the same mass of AuNS (30 μg/mL) as in **AZGL** as a comparison. **C** The temperature-increasing curves of **AZGL** (50 μg/mL) exposed to 980 nm laser irradiation for 10 min at varied power densities. **D** Heating and cooling circles for testing the stability of **AZGL** (50 μg/mL) exposed to 1.0 W/cm^2^ 980 nm laser irradiation. **E** Linear time data versus − ln(θ) obtained from the cooling period of **AZGL**

For the verification of the photothermal ability of **AZGL** (50 μg/mL), the temperature trends of its aqueous solution were recorded under irradiation with a 980 nm laser. As shown in Fig. 4A, the temperature rises from 20.0 to 60.6 °C upon irradiation as short as 5 min, indicating that such hyperthermia could induce effective tumor damage. In addition, the same mass of AuNS (30 μg/mL) as in **AZGL** (50 μg/mL) showed a similar heating effect, indicating that the formation of the nanocomposites well preserved the photothermal performance of AuNS. In addition, the gradient concentrations of 12.5, 25.0, and 50.0 μg/mL for **AZGL** gave the corresponding temperature differences (ΔT) to be 26.0, 35.1, and 48.0 °C, respectively after 10 min irradiation (Fig. 4B). The temperature rise rate of **AZGL** (50.0 μg/mL) increased significantly with the rising of the laser power (Fig. 4C) with H_2_O as control. Notably, there was no significant alteration in the photothermal effect after three cycles of heating and cooling processes (Fig. 4D). According to Roper's method [31], the photothermal conversion efficiency of **AZGL** was determined to be ca 27.5% based on the linear fitting of the data points derived from the cooling curve in Fig. 4E. Overall, **AZGL** exhibited a concentration-dependent PTT effect and excellent photo-stability, resulting in a great significance for further application in cancer treatment.

### In vitro PDT properties of AZG

**Fig. 5:**
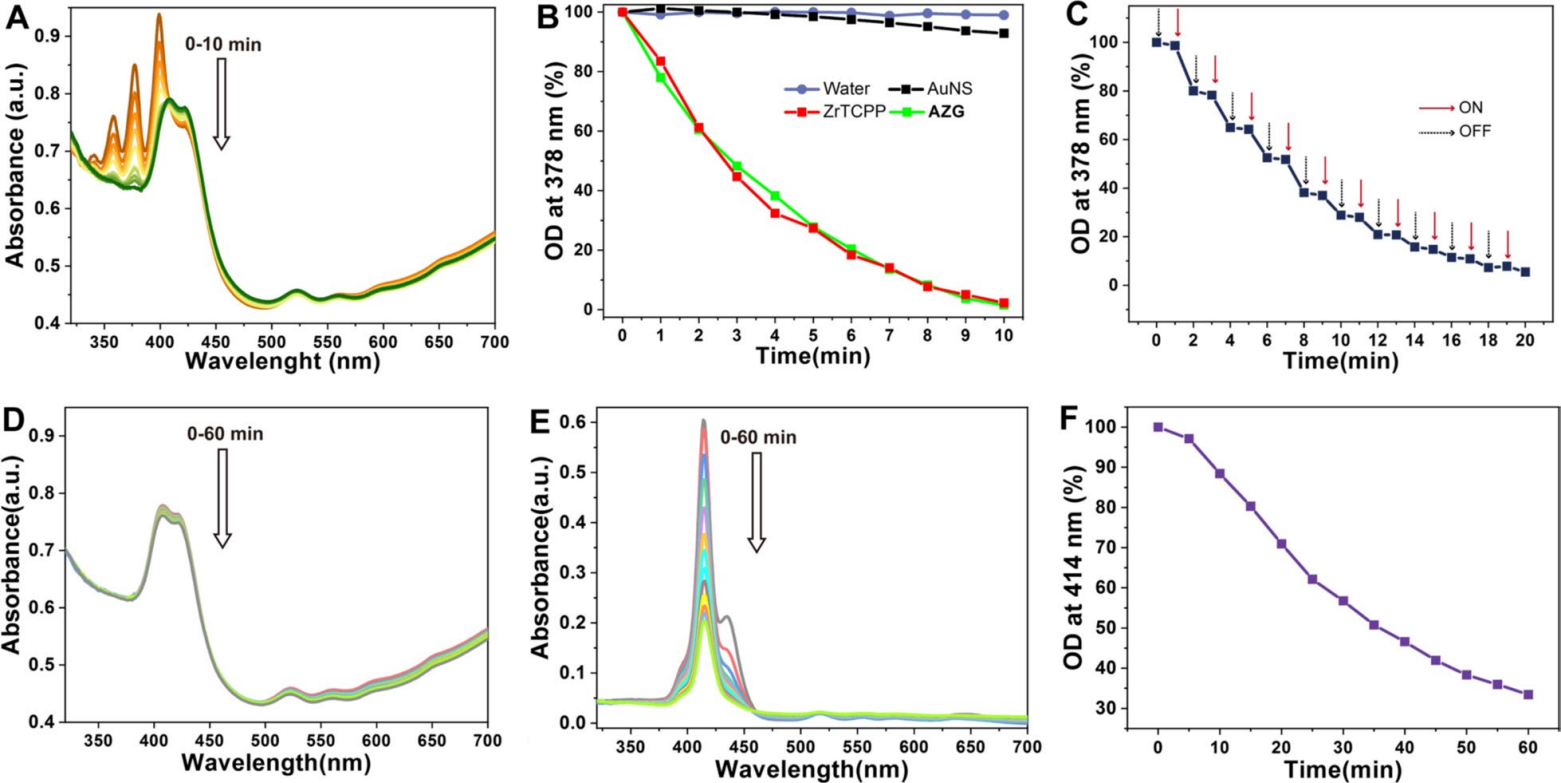
**A** Time-dependent changes of ADPA absorption caused by ^1^O_2_ treated with **AZG** under 660 nm laser irradiation. **B** The OD changes of ADPA at 378 nm with water, AuNS, ZrTCPP, and **AZG** under 660 nm laser irradiation at different time. **C** The OD changes of ADPA on the response of on–off irradiation for **AZG**. Photo-stability analysis of **AZG** (**D**) and free TCPP (**E**) under 660 nm laser irradiation for 60 min with the interval of 5 min. **F** The rate of decline in free TCPP absorption at 410 nm under 660 nm laser irradiation for 60 min with the interval of 5 min

To evaluate the photodynamic therapeutic effect, we further investigated the ^1^O_2_ production ability of AuNS, ZrTCPP, and **AZG** under 660 nm laser irradiation by monitoring the absorption of ADPA as a ROS probe at 378 nm. It is unsuitable to study the PDT for **AZGL** in vitro because the coated liposome will be oxidized by the formed ^1^O_2_ as potential interference of results [52]. As shown in Fig. 5A, the UV–Vis intensity of ADPA (λ = 378 nm) decreased by 98.47% after **AZG** (45 μg/mL) was exposed to light for 10 min, indicating its strong ^1^O_2_ generating capability. The same mass of ZrTCPP (8 μg/mL) as in **AZG** (45 μg/mL) showed similar results, signifying that the PDT effect of ZrTCPP was also well-preserved in **AZG**. Of note, pure water and AuNS had a negligible impact on ^1^O_2_ generation (Fig. 5B). In addition, the **AZG** system exhibits an on–off irradiation effect, namely, ^1^O_2_ could only be produced under the irradiation of light (Fig. 5C). Strikingly, there was no photo-bleaching effect even under 60 min irradiation for **AZG** (Fig. 4D), while for free TCPP ligand, the UV–Vis absorption decreased by 66.6% (Fig. 5E, F). Accordingly, **AZG** not only has a strong ability to generate ^1^O_2_ under laser irradiation but also has strong stability in response to light irradiation.

### Anti-immune clearance and subcellular location of AZGL

**Fig. 6:**
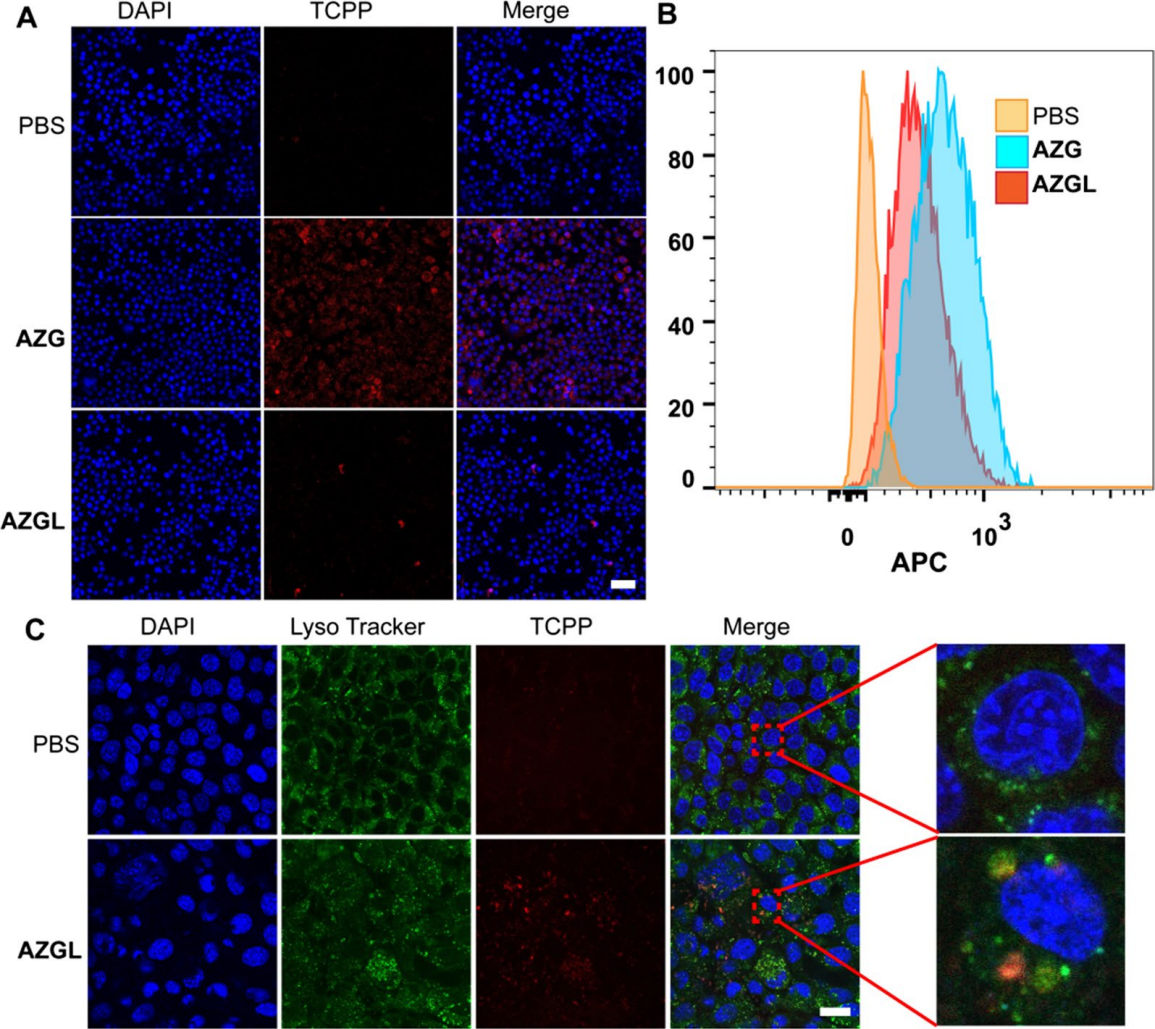
**A** The CLSM images and **B** the flow cytometry analysis of RAW264.7 after the treatment of PBS, **AZG**, and **AZGL**, respectively (Scale bar = 40 μm). **C** CLSM images of 4T1 cells with the addition of **AZGL** and staining with LysoTracker (Scale bar = 20 μm)

With the advantages of small particle size and specific surfaces, nanoparticles have been widely used in drug delivery, tumor diagnosis, and treatment through the EPR effect [53]. The EPR effect for macromolecular drug delivery to solid tumors: improvement of tumor uptake, lowering of systemic toxicity, and distinct tumor imaging in vivo [54]. However, after intravenous injection, nanoparticles can readily be recognized by the reticuloendothelial system, followed by removal with macrophages via phagocytosis. Therefore, anti-immune clearance is particularly important. PEGylated liposome exhibits anti-immune clearance and has a longer half-life in the blood due to the formation of size corona-shaped entities [55]. Therefore, we used macrophage RAW 264.7 to evaluate the anti-immune clearance of **AZGL** and **AZG**. As shown in Fig. 6A, when the cells were treated with **AZGL** and **AZG**, the red fluorescence generated from the TCPP of **AZGL** was significantly weaker than that of **AZG**. Similarly, the fluorescence intensity in the **AZGL** group analyzed by flow cytometry was also significantly lower than that of **AZG** (Fig. 6B). These results revealed that **AZGL** has great potential to escape from the immune system. In addition, the subcellular localization of **AZGL** was studied by co-localization with lysosomal fluorescent probes, which confirmed that the red fluorescent **AZGL** co-localized well with the lysosome (Fig. 6C). It could be inferred that **AZGL** is internalized into 4T1 cells through the endocytic pathway, and then accumulated in the lysosome. In the lysosome, the acidic microenvironment provided the conditions for the decomposition of **AZGL** and trigger the cascade therapies.

### The cellular uptake ability and the intracellular ROS generation ability of AZG and AZGL

**Fig. 7:**
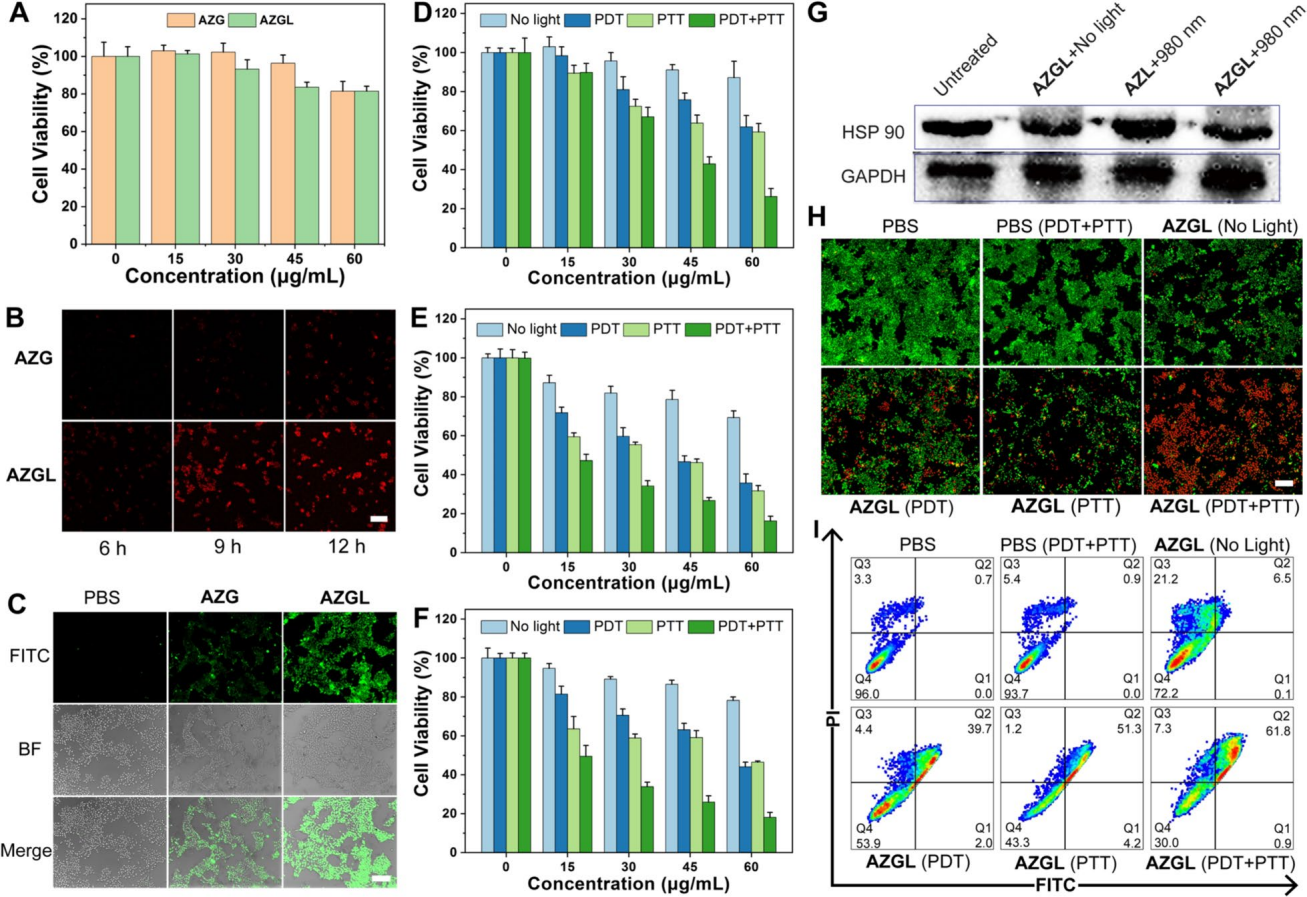
**A** The cell viability of RAW 264.7 cells with different concentrations of **AZG** and **AZGL** for 24 h. **B** The cellular uptake of **AZG** and **AZGL** at different points of time under CLSM observation (scale bar = 50 μm). **C** The generation of intracellular ROS of 4T1 cells with **AZL** and **AZGL** according to CLSM images (scale bar = 100 μm). The cell viability of 4T1 cells with different concentrations of **D** **AZL** and **E** **AZGL** (Data are means ± SD; N = 3). **F** The cell viability of MDA-MB-231 cells with different concentrations of **AZGL** (Data means ± SD; N = 3). **G** Western blot images of HSP90 expression levels with GAPDH as an internal reference for cell lysates of 4T1 cells after different treatments as indicated. **H** The live/dead staining assay of **AZGL** with various treatments using Calcein-AM/PI kit (scale bar = 200 μm). **I** Cell death assay of **AZGL** with various treatments using Annexin V/PI kit by flow cytometry analysis. *Q1* early apoptotic; *Q2* secondary necrotic/late apoptotic cells; *Q3* necrotic cells; *Q4*: vital cells

Given the superior photothermal effect and the ^1^O_2_ production performance of **AZGL** in solution, its biocompatibility was further investigated. As shown in Fig. 7A, the cell survival rates of RAW264.7 macrophages after 24 h incubation with **AZG** and **AZGL** were above 80% within 60 μg/mL, indicating their good biocompatibility with normal cells. Subsequently, we investigated the uptake ability of 4T1 cells toward **AZG** and **AZGL** with incubation time for 6, 9, and 12 h, respectively. As shown in Fig. 7B, the red fluorescence of TCPP in the cells incubated with **AZGL** gradually increased and reached saturation for about 9 h. On the contrary, **AZG** had no obvious fluorescence signal even after 12 h treatment. The DCFH-DA probe was used to detect intracellular ROS levels. As shown in Fig. 7C, the green fluorescence in cells with incubation of **AZGL** under 660 nm laser irradiation was significantly enhanced compared with **AZG**. The results of cell uptake and ROS generation experiments both proved that the nanoparticle encapsulation by liposomes could effectively facilitate the cell entry efficiency of the nanocomposite.

### The synergistic effect of PDT, PTT, and chemotherapy by AZGL

The synergistic effect of PDT, PTT, and chemotherapy by **AZGL** was conducted at the cell level. As shown in Fig. 7D, E, both **AZG** and **AZGL** showed a concentration-dependent profile and superior multimodal-therapy than mono-therapy against 4T1 cells. Due to the presence of GA in the **AZGL** nanocomposite, PTT and PDT alone, or the combination therapy showed stronger activity than the **AZL** group. For instance, in the case of **AZL** and **AZGL** at 60 μg/mL, the cell survival rates were 87.2%/69.4% for the dark control, 62.0%/35.8% for PPT, 59.3%/31.8% for PDT, 26.2%/16.3% for combined PDT and PTT, all indicating that GA has exerted a significant degree of chemotherapeutic effect against 4T1 cell lines. Similarly, **AZGL** also has a similar therapeutic effect on human breast cancer cell MDA-MB-231 (Fig. 7F).

Then, the western blot experiments were conducted for further verification of the role of GA in down-regulating heat shock protein (HSP90). As shown in Fig. 7G, **AZL** group under 980 nm laser irradiation, HSP90 was significantly up-regulated, while for **AZGL** group at the same condition, the expression of HSP90 was down-regulated. These results indicated that PTT-induced heat stress could up-regulate the expression of HSP90 in cancer cells, whereas GA has a significant inhibitory effect on PTT-induced tumor overexpression of HSP90 to promote the therapeutic effect of PTT.

To further verify the synergistic effect of treatment, Calcein-AM/PI kit was used to perform the live and dead cell staining assay. As displayed in Fig. 7H, **AZGL** (60 μg/mL) induced about 20% death of 4T1 cells without laser irradiation whereas PDT increase the cell death to 50%, and PTT treatment further induced about 60% cell death. Notably, the combination of PDT and PTT induced about 90% of cell death through destructive cell death, which was consistent with the results of the MTT experiment. In parallel with the live/dead cell staining assay, the cell death pathways of **AZGL** were further studied by flow cytometry after staining with an Annexin V/PI kit. Early apoptotic cells are those that are positive for Annexin V alone, necrotic cells are those that are positive for PI alone, and secondary necrotic and/or late apoptotic cells are those that are positive for Annexin V plus PI. As shown in Fig. 7I, the percentage of cells in the Q1, Q2 and Q3 phase was 0.1%, 6.5% and 21.2% without laser irradiation, 2.0%, 39.7% and 4.4% with PDT, 4.2%, 51.3% and 1.2% with PTT, 0.9%, 61.8% and 7.3% with both PTT and PDT. These experiments showed that the combination of PDT and PTT induced stronger secondary necrotic/late apoptotic cells. While PDT or PTT alone causes weaker secondary necrotic/late apoptotic cells.

### In vivo antitumor performance of AZGL

A severe hemolytic side effect is the main obstacle hindering the clinical application of anticancer drugs [56]. Thus, the hemolytic assay was conducted in determining the biocompatibility and the feasibility of intravenous injection of **AZGL**. The results showed that **AZGL** demonstrated almost no hemolytic effect even at the high concentration of 200 µg/mL (Additional file 1: Figure S8). The good blood compatibility proved that **AZGL** is a safe nanomaterial for intravenous administration.

**Fig. 8:**
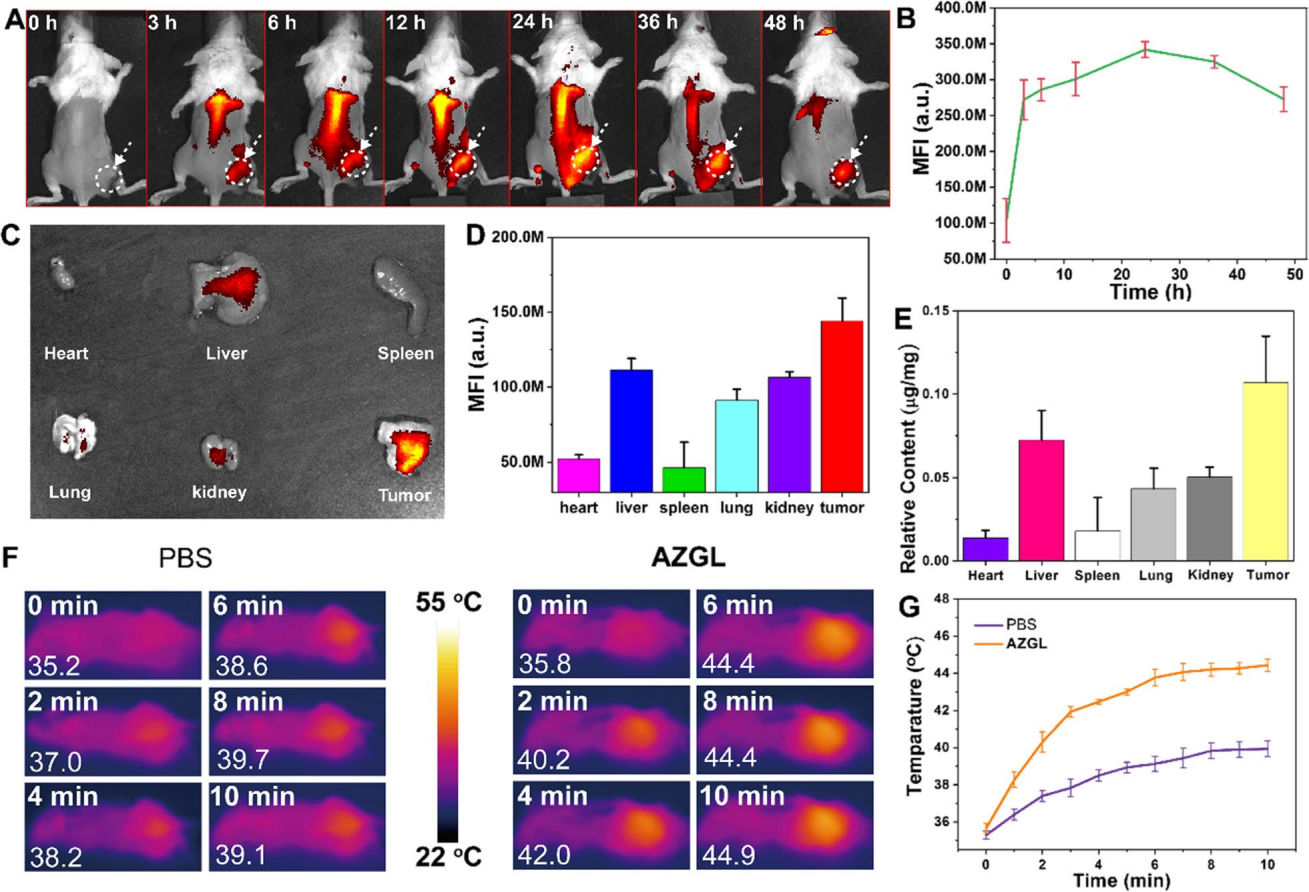
**A** The in vivo fluorescence images of the mice and **B** mean fluorescence curve of tumor site at different times after intravenous injection of **AZGL**. **C** Ex vivo fluorescence imaging and **D** mean fluorescence intensity of tumor and major organs after intravenous injection of **AZGL**. **E** The relative content of Au in tumors and major organs from mice treated after intravenous injection of **AZGL**. **F** The in vivo infrared thermal images of the mice and **G** the temperature change curves of tumor site after intravenous injection of PBS and **AZGL** with 980 nm laser irradiation for different periods from 0 to 10 min

To visualize and verify the accumulation feasibility of **AZGL** at the tumor site, a female BALB/c mouse model was established for estimating the bio-distribution of **AZGL** on a small-animal imaging system. As expected, the fluorescence intensity of the tumor region continuously increased and reached the maximum value at 24 h post-injection (Fig. 8A, B). Even at 24 h post-injection, the fluorescence was observed throughout most parts of the body of the mice, suggesting the long blood circulation time could be especially advantageous to exert the EPR effect. Therefore, the best time point of laser irradiation for PDT and/or PTT was conducted at 24 h post-injection. After 24 h, the mice were sacrificed to extract the tumors and main organs for imaging. As shown in Fig. 8C, D, **AZGL** is effectively accumulated and retained in tumor tissues via the EPR effect, which may attribute to its appropriate size and the coated LP that engender its stability at physiological conditions [57]. For further evidence of the bio-distribution of **AZGL**, the amount of gold (Au) in major organs (heart, liver, spleen, lung, kidney) and the tumor tissues were quantitatively assessed by inductively coupled plasma mass spectrometry (ICP-MS). As shown in Fig. 8E, Au can effectively accumulate and retain in tumor tissues, which is in line with the quantitative analysis of fluorescence imaging results.

The light-triggered temperature changes of **AZGL** were monitored using an infrared thermography camera at 24 h post-injection. As shown in Fig. 8F, G, the temperature of the **AZGL** group in tumor position increased from 35.8 to 44.9 °C after 10 min of irradiation, while that of the PBS group only increased from 35.2 to 39.1 °C. These results indicated that **AZGL** possesses favorable accumulation in tumor position to exert a mild photothermal effect.

**Fig. 9:**
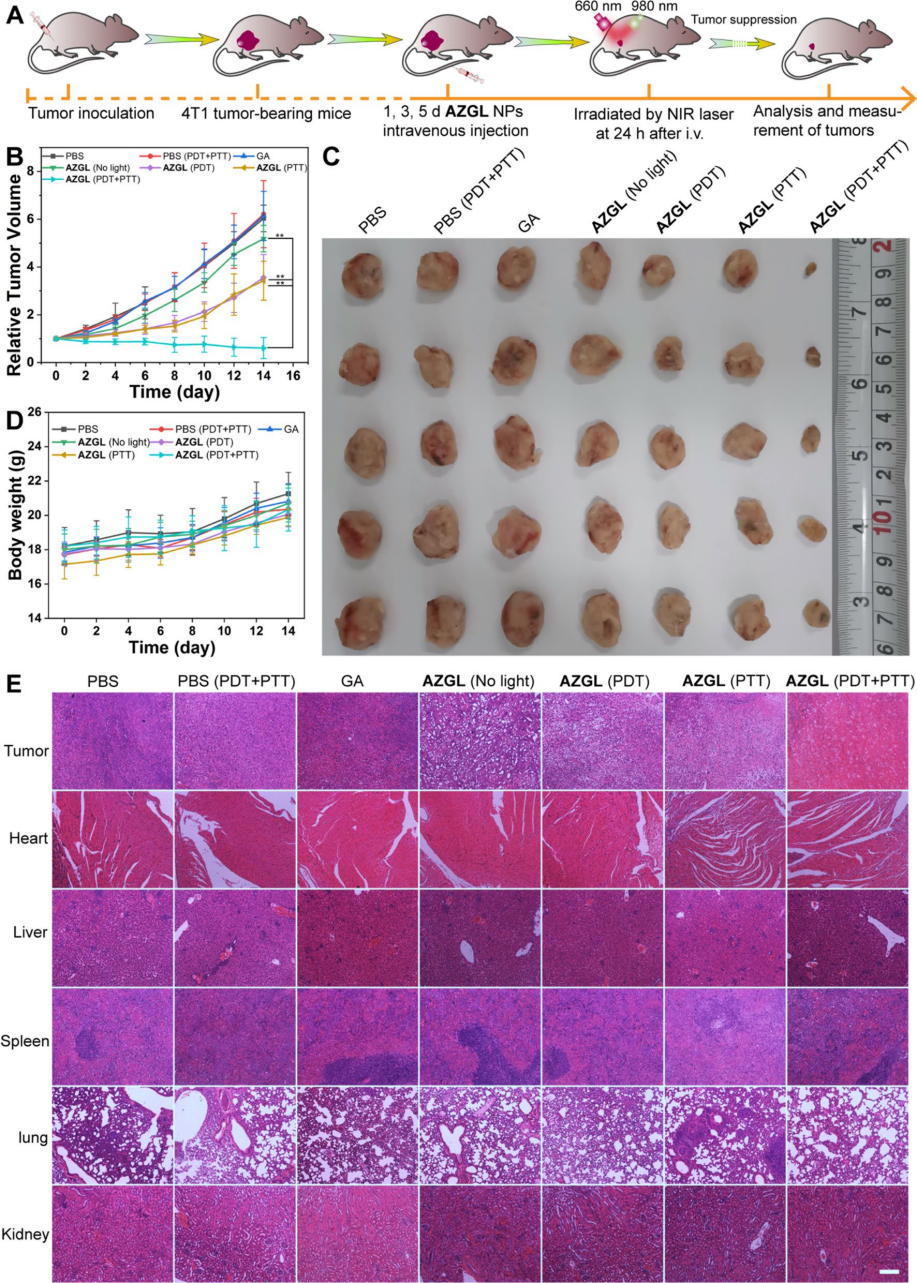
The in vivo antitumor performance (***p < 0.001, **p < 0.01, or *p < 0.05 were calculated by a Student’s t-test). **A** Schematic illustration of in vivo NIR light-triggered combinational therapy procedure on mice. **B** Tumor growth curves in different groups (the tumor volume was relative to their initial sizes). **C** Tumor images in different groups were obtained on the 14th day. **D** The body weight changes in different groups. **E** H&E staining of main organs (tumor, heart, liver, spleen, lung, and kidney) tissue slices after treatments (on the 14th day) for different groups (scale bar = 200 μm)

Given the above-mentioned favorable blood compatibility and the tumor accumulation of **AZGL**, we aimed to carry out the combined therapy on 4T1 tumor-bearing mice. The mice were randomly divided into 7 groups (5 in each group), namely, PBS, PBS (PDT + PTT), GA, **AZGL** (No light), **AZGL** (PDT), **AZGL** (PTT), and **AZGL** (PDT + PTT). This therapeutic regimen was executed on the 1st, 3rd, and the 5th day and the light irritation was conducted at 24 h after intravenous injection (Fig. 9A). As shown in Fig. 9B, the tumor size of the control group (PBS and PBS with PDT and PTT) increased rapidly over time and the trend of the GA group also found to be similar. Likely, GA is not able to reach the tumor site by intravenous injection to inhibit tumor growth probably due to the quick clearance [58]. Meanwhile, a certain inhibition of tumor growth was observed in the **AZGL**, **AZGL** (PDT), and **AZGL** (PTT) groups due to their suitable size and anti-immune clearance as discussed above, with the **AZGL** (PDT + PTT) group also featuring the best therapeutic efficacy and the tumor almost disappeared upon treatment. After 14 days, all mice were sacrificed, and tumor tissues were dissected and weighed. As shown in Fig. 9C and Additional file 1: Figure S9, the photos of the tumors extracted from the mice exhibited apparent tumor suppression in the **AZGL** (PDT + PTT) group than the other control groups, which was consistent with the tumor growth curves. As shown in Fig. 9D, the bodyweight of all mice in the seven groups did not change significantly, which demonstrated the biological safety of **AZGL** in vivo. The biological safety of **AZGL** was further supported by the blood biochemistry analysis of serum (AST, ALT, APK, and BUN) performed after different treatments (Additional file 1: Figure S10).

H&E staining of the main organs (heart, liver, spleen, lung, and kidney) indicated that there was no obvious damage or necrosis in the normal tissues such as inflammatory response and necrosis during the treatment process (Fig. 9E). These results support that the nanocomposite of **AZGL** possessed favorable biocompatibility for combination therapy in vivo. In stark contrast, the tumor tissue had the largest degree of destructive cell necrosis in group **AZGL** (PDT + PTT), which was in line with the above results.

**Fig. 10:**
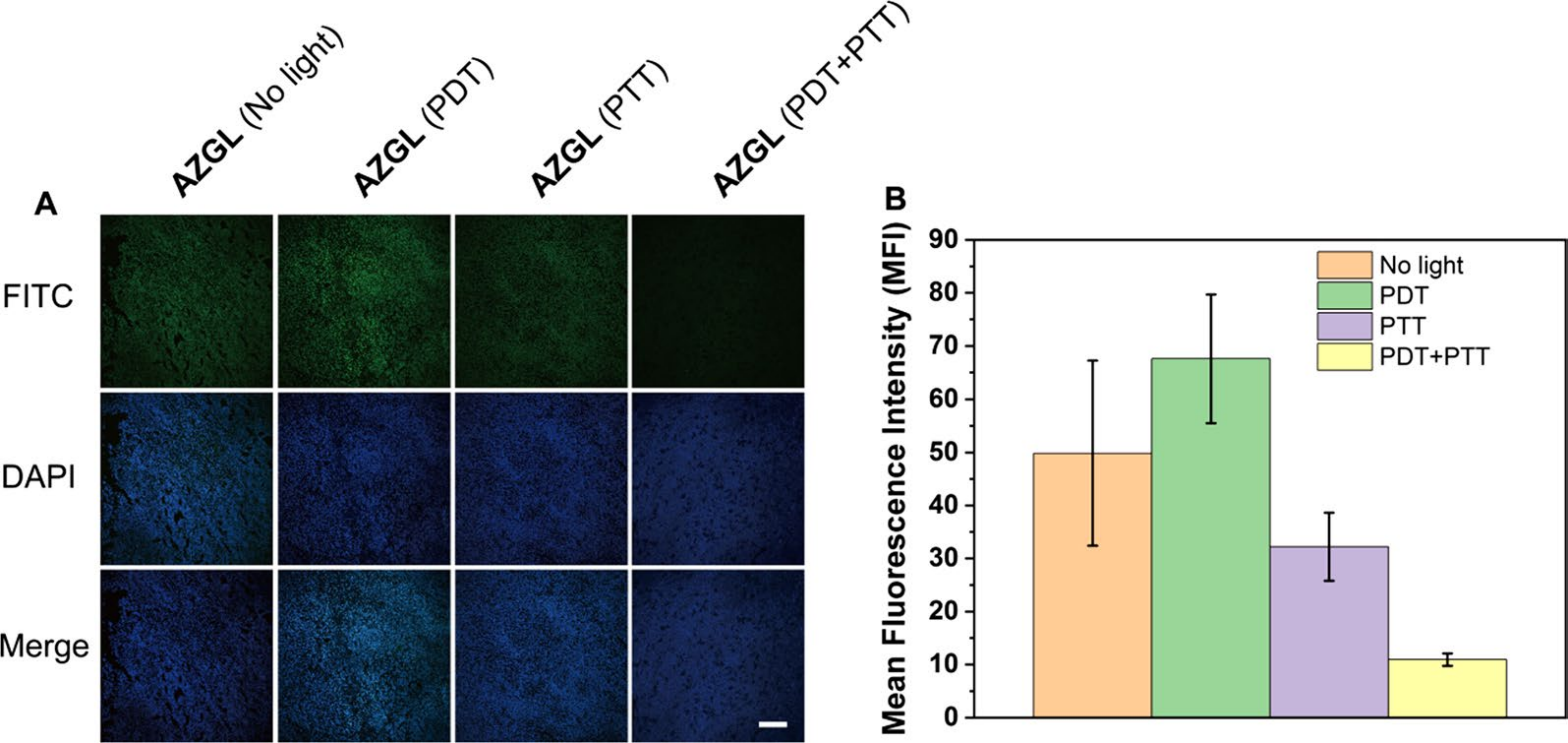
**A** CLSM images and **B** quantitative analysis of the fluorescence of tumor hypoxic evaluation after **AZGL** administrations before and after laser irradiation. The nucleus was stained by DAPI (blue) and the hypoxia site was stained with HIF-1α antibody (green) (scale bar = 200 μm)

To further explore the internal mechanism of this significant synergistic effect, a hypoxia-inducible factor (HIF-1α, a typical marker of hypoxia) staining assay was performed to evaluate the hypoxia level in the tumor. As shown in Fig. 10A, B, compared with the **AZGL** (dark control) group, the tumor tissue of the **AZGL** (PDT) group showed a stronger green fluorescence signal, meanwhile, the **AZGL** (PTT) group showed a weaker green fluorescence signal, demonstrating aggravated hypoxia in the tumor by PDT, and relatively relieved hypoxia by PTT. Moreover, the **AZGL** (PDT + PTT) group showed the weakest green fluorescence signal due to the destruction of tumor cells after the combined therapy [17]. These results confirmed the findings that **AZGL** is activated by light for PTT to alleviate the hypoxic environment in tumors for improving the effect of PDT, resulting in the accelerated activation of GA for the HSP90-inhibitory activity, and finally achieving the synergistic antitumor effect.

## Conclusion

In summary, we have prepared a core–shell nanocomposite **AZGL** that integrated AuNS-mediated mild PTT, TCPP-mediated PDT, and GA-mediated chemotherapy for the in vitro and in vivo treatment of breast cancer. The prepared **AZGL** nanocomposite can effectively generate ROS under 660 nm laser radiation, thereby killing heat-resistant tumor cells. On the other hand, the heat generated by the photothermal effect of **AZGL** can not only kill the cancer cells but also relieve the hypoxic environment of solid tumors and further enhance the PDT effect. Furthermore, GA molecules exert the chemotherapeutic effect meanwhile block HSP90 overexpressed by heat stress to re-sensitize PTT. In general, the strategy designed in this study has the following advantages: (a) the designed **AZGL** nanocomposite can induce effective apoptosis/necrosis of cancer cells under mild hyperthermia, which can not only minimize non-specific heat to normal tissues injury but also achieve effective photothermal treatment of deep tumors; (b) **AZGL** nanocomposite is sensitive to the acidic microenvironment of solid tumors, to realize its tumor-specific drug release and localization; (c) **AZGL** nanocomposite have good accumulation in tumor tissues, which significantly improves their imaging and treatment performance. The versatile theranostic nanoplatform presented herein demonstrated tremendous advantages and potential applications for clinically precise theranostics of the most solid tumors and hypoxia-associated diseases.

## Supplementary Information

**Additional file 1: Figure S1.** **A** UV spectrum and **B** curve of different molar concentrations (Zr^4+^=TCPP) at 410 nm. **Figure S2.** EDS spectrum of AuNS. **Figure S3.** **A** PXRD analysis of ZrTCPP, AuNS, and **AZG**. **B** Nitrogen (N_2_) adsorption-desorption isotherms at 77K. **Figure S4.** Cell viability of RAW264.7 cells with different concentrations of GA. Data are presented by means ± SD with n = 3. **Figure S5.** The standard curve of **A** GA (361 nm) and the HPLC of GA **B**. **C** TCPP (410 nm) and **D** The standard curves of AuNS (980 nm). **Figure S6. **The Fourier transform infrared (FT-IR) spectroscopy of AuNS, GA, TCPP, **AZ **and **AZG**. **Figure S7. A** The Full XPS of **AZG**. **B** The XPS of Zr3d and **C** N1s spectrum of **AZG**. **Figure S9. **Tumor weight in different groups obtained on the 14th day (***p < 0.001, **p < 0.01, or *p < 0.05 were calculated by a Student’s t test). **Figure S10.** In vivo biological safety evaluated by biochemical analysis of serum after the different treatments (n = 3).

## Data Availability

All data generated or analyzed during this study are included in this published article and the additional information.

## Abbreviations

GA: Gambogic acid
HSP90: Heat-shock protein 90
AuNS: Gold nanostars
TCPP: Tetrakis (4-carboxyphenyl) porphyrin
LP: PEGylated-modified liposome
NMOF: Nanoscale metal−organic framework
PTT: Photothermal therapy
PDT: Photodynamic therapy
ROS: Reactive oxygen species
NIR: Near-infrared
TDSP-Exos: Tumor cell-derived stellate plasmonic exosomes
ZrOCl_2_·8H_2_O: Zirconyl chloride octahydrate
PVP: Pyrrolidone
ADPA: 9,10-Anthracenedipropionic acid
DCFH-DA: 2′,7′-Dichlorofluorescin diacetate
MTT: Methyl-thiazolyl diphenyl-tetrazolium bromide
DAPI: 4′,6-Diamidino-2-phenylindole
DSPC: 1,2-Distearoyl-*sn*-glycero-3-phosphocholine
DSPE-PEG-COOH: 2-Distearoyl-*sn*-glycero-3-phosphoethanolamine-N-[carboxy(polyethylene glycol)-2000]
PVDF: Polyvinylidene difluoride
UV–Vis: Ultraviolet–visible
FT-IR: Fourier-transform infrared spectroscopy
PXRD: Powder X-ray diffraction
XPS: X-ray photoelectron spectroscopy
ICP-MS: Inductively coupled plasma-mass spectrometry
TEM: Transmission electron microscope
CLSM: Confocal laser scanning microscopy
HPLC: High-performance liquid chromatography
HAuCl_4_: Chloroauric acid
AgNO_3_: Silver nitrate
AA: Ascorbic acid
DI water: Deionized water
EE: Encapsulation efficiency
DL: Loading capacity
DFT: Density functional theory
B3LYP: Becke’s three-parameter hybrid
SDD: Stuttgart/Dresden
HOMO: Highest occupied molecular orbital
LUMO: Lowest unoccupied molecular orbital
OD: Optical density
^1^O_2_: Singlet oxygen
HRP: Horseradish peroxidase
GAPDH: Glyceraldehyde-3-phosphate dehydrogenase
RBCs: Red blood cells
H&E: Hematoxylin and eosin
HIF-1α: Hypoxia-inducible factor-1α
FRET: Fluorescence resonance energy transfer
DLS: Dynamic light scattering
DMEM: Dulbecco’s modified eagle medium
