# Supplementary material for: Synergistic photothermal-photodynamic-chemotherapy toward breast cancer based on a liposome-coated core–shell AuNS@NMOFs nanocomposite encapsulated with gambogic acid
Source: J Nanobiotechnology. 2022 May 6;20:212. doi: 10.1186/s12951-022-01427-4 (PMC9074336; doi:10.1186/s12951-022-01427-4)
Supplement: Supplementary file 1 — Additional file 1: Figure S1. A UV spectrum and B curve of different molar concentrations (Zr4+=TCPP) at 410 nm. Figure S2. EDS spectrum of AuNS. Figure S3. A PXRD analysis of ZrTCPP, AuNS, and AZG. B Nitrogen (N2) adsorption-desorption isotherms at 77K. Figure S4. Cell viability of RAW264.7 cells with different concentrations of GA. Data are presented by means ± SD with n = 3. Figure S5. The standard curve of A GA (361 nm) and the HPLC of GA B. C TCPP (410 nm) and D The standard curves of AuNS (980 nm). Figure S6. The Fourier transform infrared (FT-IR) spectroscopy of AuNS, GA, TCPP, AZ and AZG. Figure S7. A The Full XPS of AZG. B The XPS of Zr3d and C N1s spectrum of AZG. Figure S9. Tumor weight in different groups obtained on the 14th day (***p < 0.001, **p < 0.01, or *p < 0.05 were calculated by a Student’s t test). Figure S10. In vivo biological safety evaluated by biochemical analysis of serum after the different treatments (n = 3). [file 12951_2022_1427_MOESM1_ESM.doc]

**Synergistic photothermal-photodynamic-chemotherapy toward breast cancer based on a liposome-coated core-shell AuNS@NMOFs nanocomposite encapsulated with gambogic acid**

Rong-Tian Li^1^, Yi-Dan Zhu^1^, Wen-Ya Li^3^, Ying-Ke Hou^1,3^, Yi-Ming Zou^1^, Ying-Hua Zhao^3^, Quan Zou^3^, Wen-Hua Zhang^2^, Jin-Xiang Chen^1^*

*Correspondence: jxchen@smu.edu.cn

^1^NMPA Key Laboratory for Research and Evaluation of Drug Metabolism, Guangdong Provincial Key Laboratory of New Drug Screening, School of Pharmaceutical Sciences, Southern Medical University, Guangzhou 510515, People’s Republic of China

^2^College of Chemistry, Chemical Engineering and Materials Science, Soochow University, Suzhou 215123, People’s Republic of China

^3^Department of Medical Imaging, Third Affiliated Hospital of Southern Medical University (Academy of Orthopedics Guangdong Province), Southern Medical University, Guangzhou, 510630, People’s Republic of China


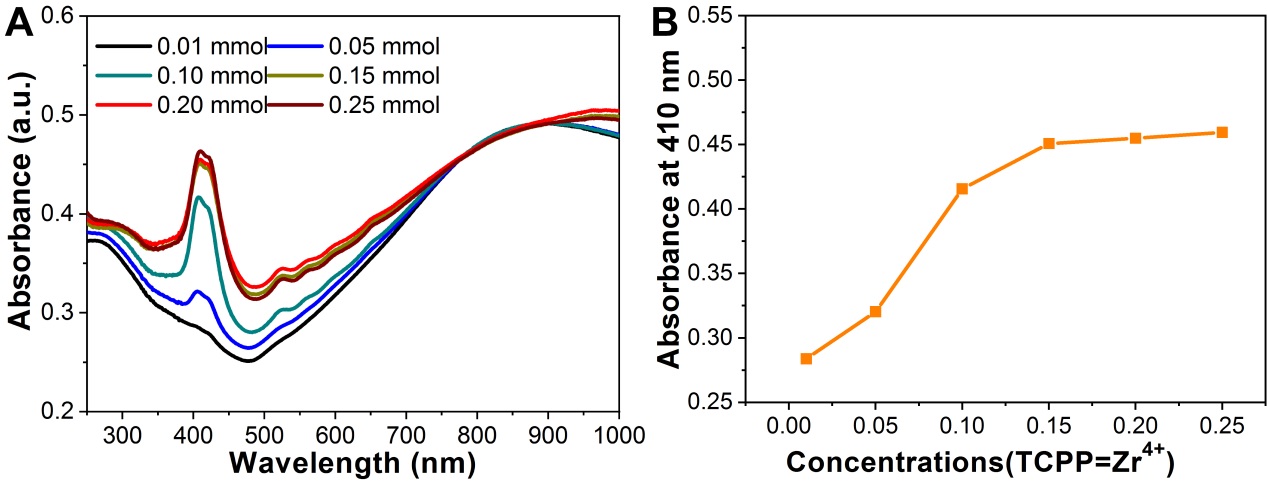


**Figure S1.** (A) UV spectrum and (B) curve of different molar concentrations (Zr^4+^=TCPP) at 410 nm.


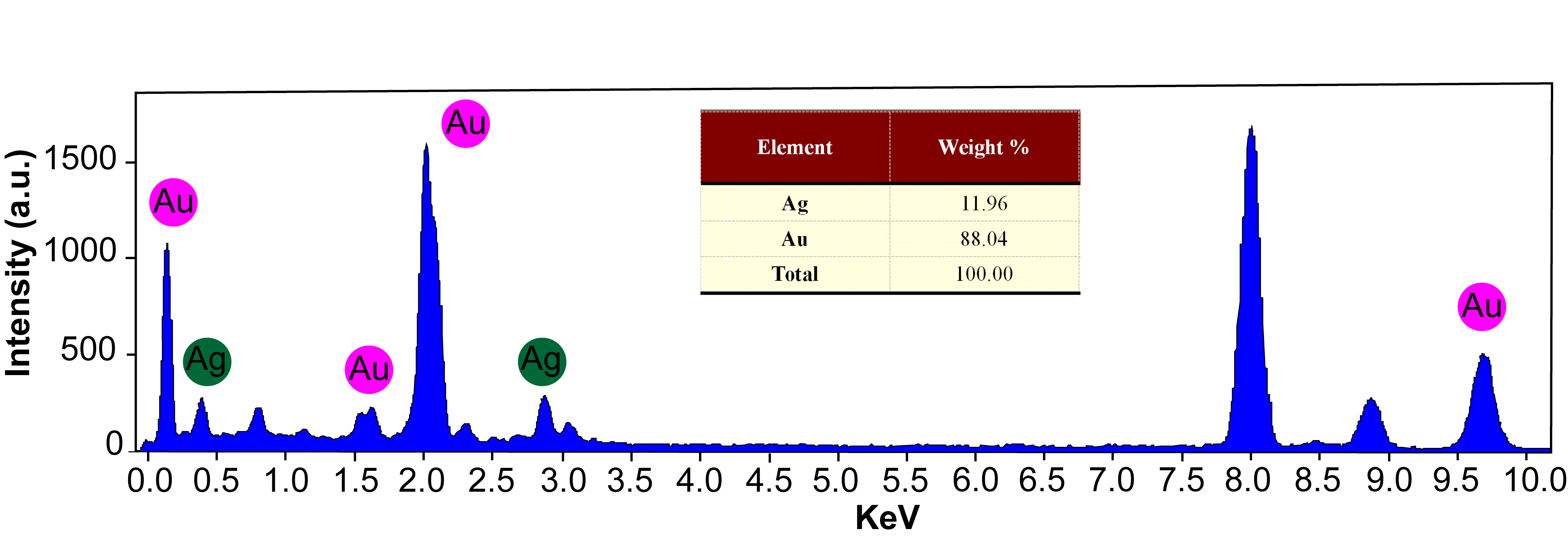


**Figure S2.** EDS spectrum of AuNS.


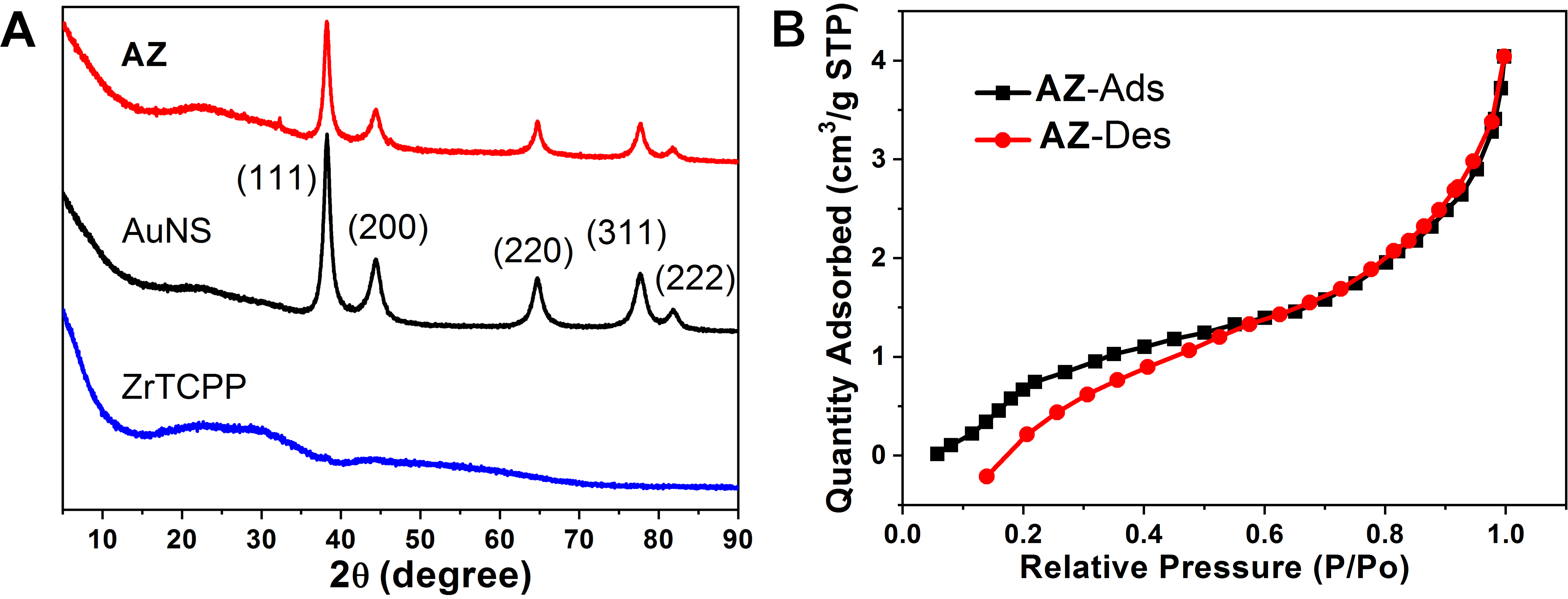


**Figure S3.** (A) PXRD analysis of ZrTCPP, AuNS, and **AZG**. (B) Nitrogen (N_2_) adsorption-desorption isotherms at 77K.


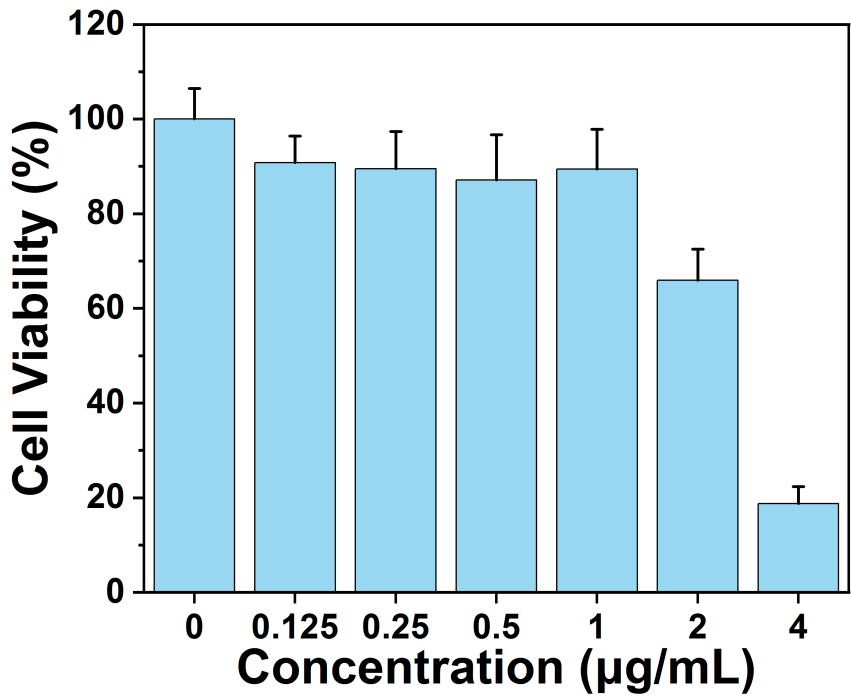


**Figure S4.** Cell viability of RAW264.7 cells with different concentrations of GA. Data are presented by means ± SD with n =3.

**
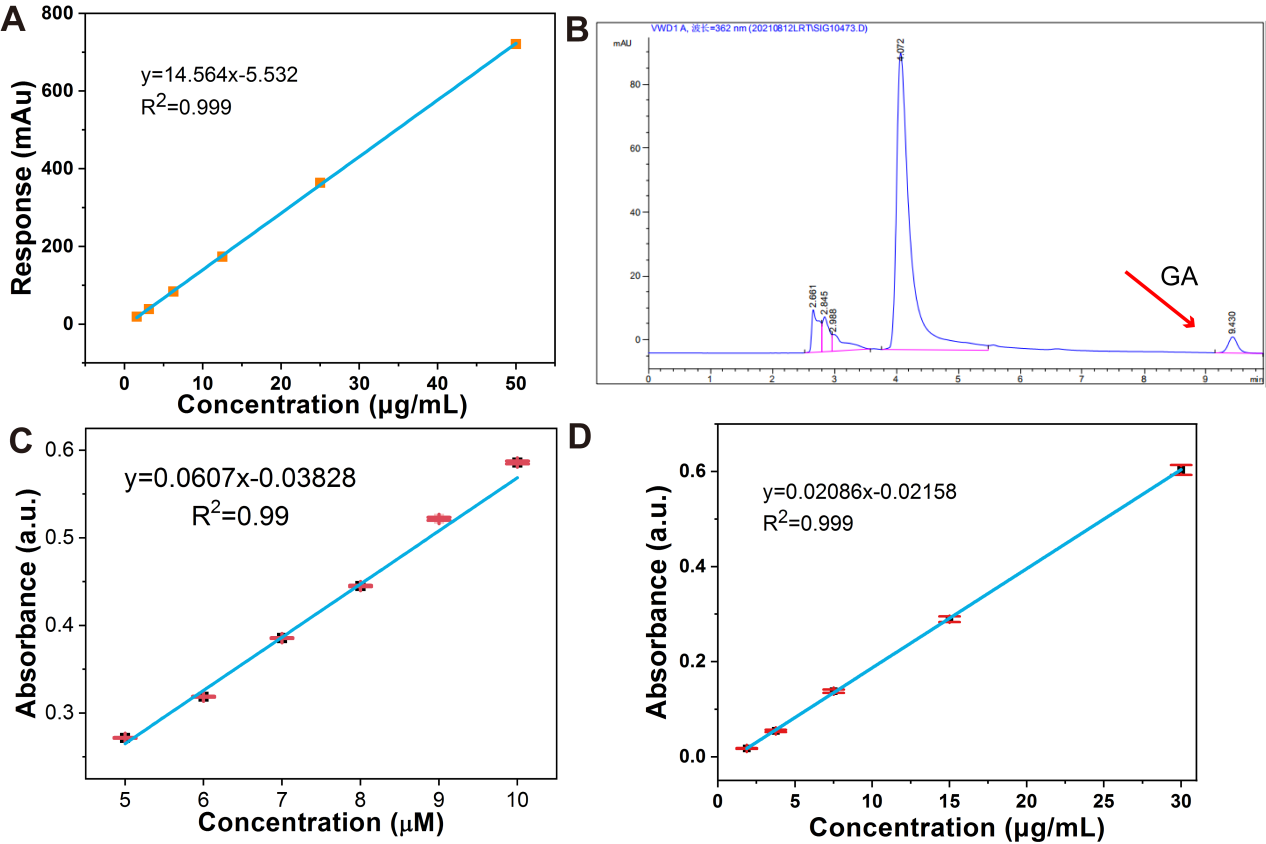
**

**Figure S5.** The standard curve of (A) GA (361 nm) and the HPLC of GA (B). (C) TCPP (410 nm) and (D) The standard curves of AuNS (980 nm).


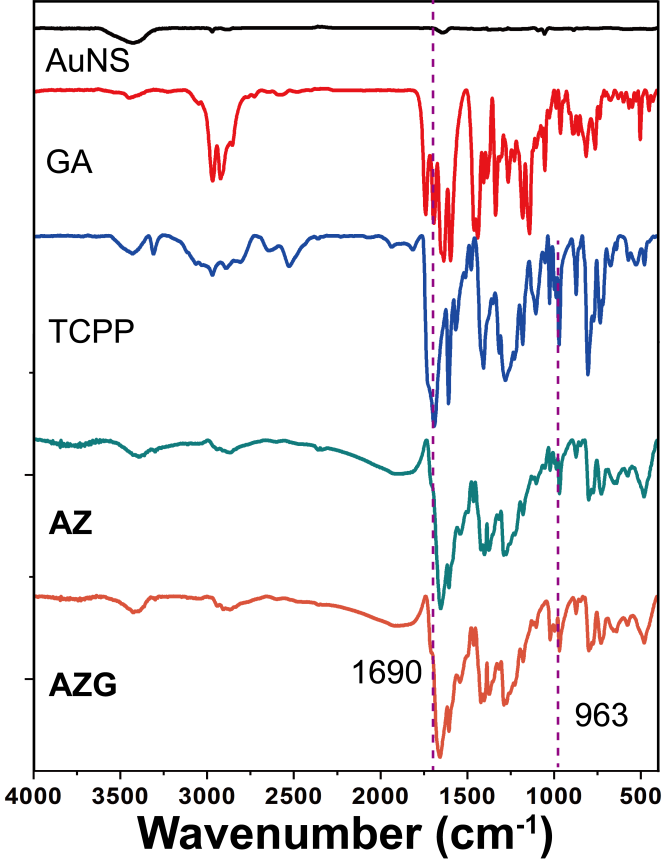


**Figure S6.** The Fourier transform infrared (FT-IR) spectroscopy of AuNS, GA, TCPP, **AZ** and **AZG**.

**A
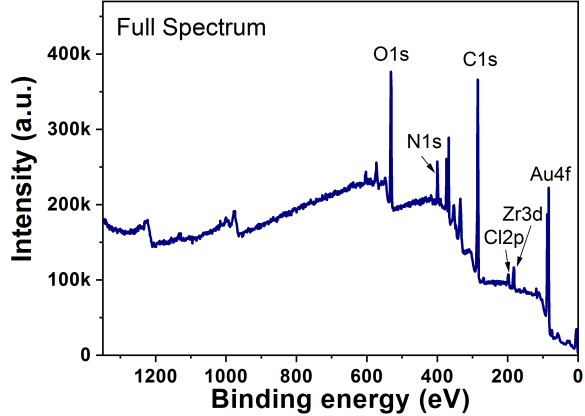
B
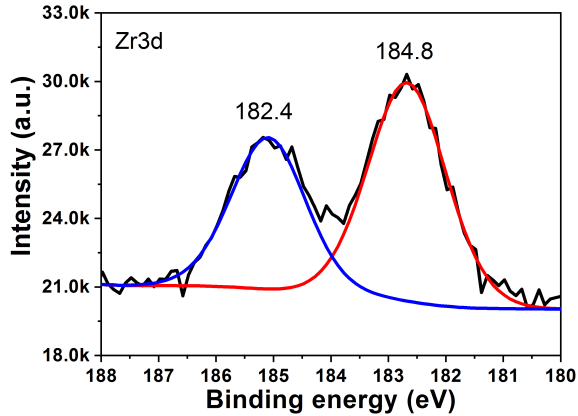
**

**C
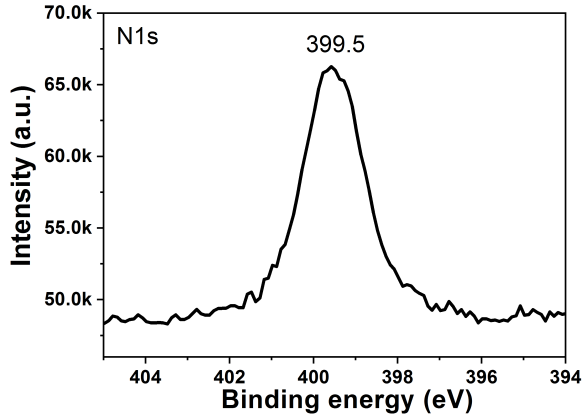
**

**Figure S7.** (A) The Full XPS of **AZG**. (B) The XPS of Zr3d and (C) N1s spectrum of **AZG**.


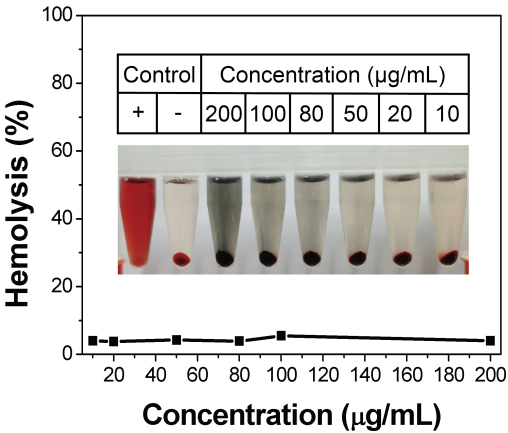


**Figure S8.** The hemolytic activity of **AZGL**.


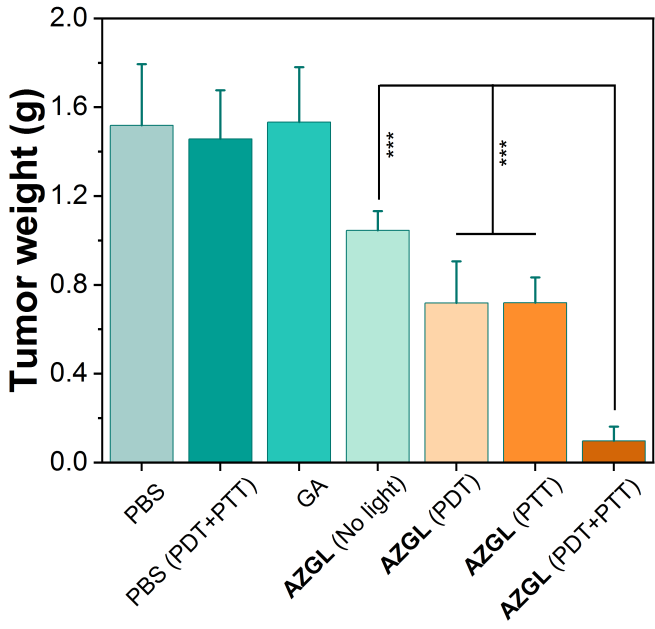


**Figure S9.** Tumor weight in different groups obtained on the 14^th^ day (***p < 0.001, **p < 0.01, or *p < 0.05 were calculated by a Student's t test).


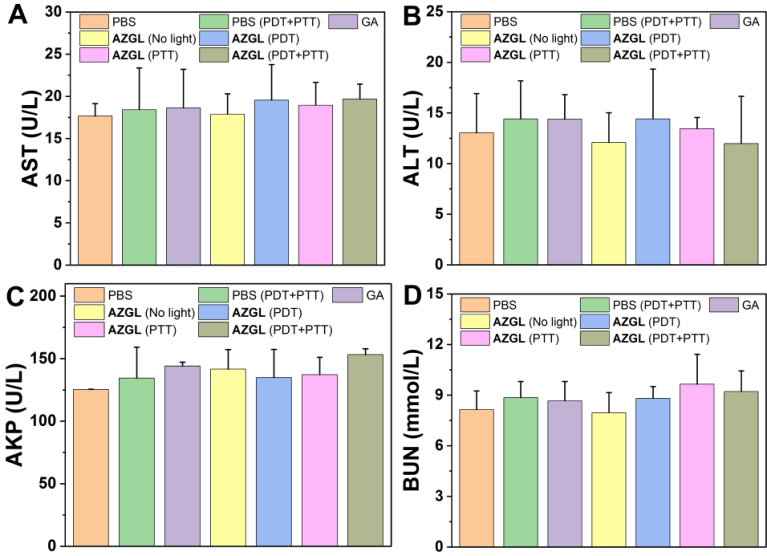


**Figure S10.** *In vivo* biological safety evaluated by biochemical analysis of serum after the different treatments (n = 3).
